# Effects, equity, and cost of school-based and community-wide treatment strategies for soil-transmitted helminths in Kenya: a cluster-randomised controlled trial

**DOI:** 10.1016/S0140-6736(18)32591-1

**Published:** 2019-05-18

**Authors:** Rachel L Pullan, Katherine E Halliday, William E Oswald, Carlos Mcharo, Emma Beaumont, Stella Kepha, Stefan Witek-McManus, Paul M Gichuki, Elizabeth Allen, Tom Drake, Catherine Pitt, Sultani H Matendechero, Marie-Claire Gwayi-Chore, Roy M Anderson, Sammy M Njenga, Simon J Brooker, Charles S Mwandawiro

**Affiliations:** a Faculty of Infectious and Tropical Diseases, London School of Hygiene & Tropical Medicine, London, UK; b Faculty of Epidemiology and Public Health, London School of Hygiene & Tropical Medicine, London, UK; c Faculty of Public Health and Policy, London School of Hygiene & Tropical Medicine, London, UK; d Eastern and Southern Africa Centre of International Parasite Control, Kenya Medical Research Institute, Nairobi, Kenya; e Pwani University Bioscience Research Centre, Pwani University, Kilifi, Kenya; f Neglected Tropical Diseases Unit, Division of Communicable Disease Prevention and Control, Ministry of Health, Nairobi, Kenya; g Deworm the World Initiative, Evidence Action, Nairobi, Kenya; h Faculty of Medicine, Department of Infectious Disease Epidemiology, London Centre for Neglected Tropical Disease Research, School of Public Health, St Mary's Campus, Imperial College London, London, UK

## Abstract

**Background:**

School-based deworming programmes can reduce morbidity attributable to soil-transmitted helminths in children but do not interrupt transmission in the wider community. We assessed the effects of alternative mass treatment strategies on community soil-transmitted helminth infection.

**Methods:**

In this cluster-randomised controlled trial, 120 community units (clusters) serving 150 000 households in Kenya were randomly assigned (1:1:1) to receive albendazole through annual school-based treatment targeting 2–14 year olds or annual or biannual community-wide treatment targeting all ages. The primary outcome was community hookworm prevalence, assessed at 12 and 24 months through repeat cross-sectional surveys. Secondary outcomes were *Ascaris lumbricoides* and *Trichuris trichiura* prevalence, infection intensity of each soil-transmitted helminth species, and treatment coverage and costs. Analysis was by intention to treat. This trial is registered with ClinicalTrials.gov, number NCT02397772.

**Findings:**

After 24 months, prevalence of hookworm changed from 18·6% (95% CI 13·9–23·2) to 13·8% (10·5–17·0) in the annual school-based treatment group, 17·9% (13·7–22·1) to 8·0% (6·0–10·1) in the annual community-wide treatment group, and 20·6% (15·8–25·5) to 6·2% (4·9–7·5) in the biannual community-wide treatment group. Relative to annual school-based treatment, the risk ratio for annual community-wide treatment was 0·59 (95% CI 0·42–0·83; p<0·001) and for biannual community-wide treatment was 0·46 (0·33–0·63; p<0·001). More modest reductions in risk were observed after 12 months. Risk ratios were similar across demographic and socioeconomic subgroups after 24 months. No adverse events related to albendazole were reported.

**Interpretation:**

Community-wide treatment was more effective in reducing hookworm prevalence and intensity than school-based treatment, with little additional benefit of treating every 6 months, and was shown to be remarkably equitable in coverage and effects.

**Funding:**

Bill & Melinda Gates Foundation, the Joint Global Health Trials Scheme of the Medical Research Council, the UK Department for International Development, the Wellcome Trust, and the Children's Investment Fund Foundation.

## Introduction

In 2012, the London Declaration on neglected tropical diseases announced a cross-sectoral commitment to control or eliminate ten neglected tropical diseases by 2020, on the basis of WHO roadmap targets.^1^ For soil-transmitted helminths (which include *Ascaris lumbricoides, Trichuris trichiura*, and the hookworms *Ancylostoma duodenale* and *Necator americanus*), the target is to provide regular anthelmintic treatment to at least 75% of children aged 1–14 years in districts where prevalence of any soil-transmitted helminth infection exceeds 20% in schoolchildren, with a view to controlling the morbidity associated with infection.^2^ By 2016, school-based deworming programmes had reached 69·5% of these children, with 33 of the 102 countries requiring preventive chemotherapy exceeding the 75% coverage target.^3^ Building upon this progress, national programmes and policy makers are now looking beyond 2020 and towards goals focused on reducing transmission. Mathematical models suggest that community-wide treatment can interrupt soil-transmitted helminth transmission^4^, ^5^ and a meta-analysis suggests it would be more effective than school-based treatment in reducing infection among school-age children.^6^

Other neglected tropical disease programmes, including those against onchocerciasis, trachoma, and lymphatic filariasis, have achieved treatment of entire communities with community health workers or volunteers, using central point or house-to-house delivery models. In addition to providing a platform for reducing community infection levels, these programmes might provide an important gateway to universal health coverage, through ensuring broad, equitable access to basic health services, particularly among the most marginalised populations.^7^, ^8^ For this reason, neglected tropical disease treatment coverage has been included as an equity tracer for the Sustainable Development Goal target 3.8 (achieving universal health coverage).^9^ However, evidence of the equity of mass drug administration programmes within targeted communities is insufficient, both in terms of coverage and effects on infection outcomes.

**Research in context**

**Evidence before this study**

The focus of control efforts for soil-transmitted helminths is annual school-based deworming, but mathematical modelling suggests that this strategy is unlikely to interrupt transmission, necessitating ongoing investment in control. The models suggest that interruption of soil-transmitted helminth transmission might be feasible if treatment is expanded to adults and provided more frequently, but empirical support of this hypothesis is scarce. A systematic review and meta-analysis suggested that community-wide treatment is more effective than school-based treatment in reducing infection among school-age children, and a non-randomised community trial of biannual mass treatment of all ages showed notable reductions in the prevalence of soil-transmitted helminth infection. However, available studies do not have adequate control groups and randomisation, have not been implemented at scale or in programmatic settings, and are under-powered.

**Added value of this study**

Our trial used a cluster-randomised design and included 120 randomly assigned community units, all of which were included in the analysis. We found that community-wide treatment with albendazole was more effective in reducing the prevalence and intensity of hookworm among all ages than school-based treatment, although biannual treatment had little additional benefit. Additionally, we investigated the socioeconomic and demographic equity of the intervention and found comparable coverage and effects of the interventions across important demographic and socioeconomic subgroups. Community-based mass drug administration is also used in other neglected tropical diseases, including lymphatic filariasis, onchocerciasis, and trachoma, which collectively reach more than 1 billion people annually. Our results are some of the first data highlighting the remarkable equity of the neglected tropical disease delivery platform within targeted communities.

**Implications of all the available evidence**

Our results are consistent with those from previous research on mathematical models of soil-transmitted helminth transmission and treatment and highlight a role for community-wide treatment in reducing prevalence and intensity of hookworm infection. These findings are highly relevant to ongoing discourse concerning post 2020 WHO goals for soil-transmitted helminths, as they highlight the potential of including a transmission goal in some settings. Our finding of the equity of the intervention illustrates the reach and equity of the neglected tropical disease delivery platform and the potential to leverage this platform to deliver other health interventions among the poorest, most marginalised communities, and thus contribute towards universal health-care coverage.

We did a cluster-randomised trial on the Kenyan coast to evaluate and compare the effects, equity, and cost of annual school-based treatment, annual community-wide treatment, and biannual community-wide treatment in reducing the prevalence and intensity of soil-transmitted helminth infection.

## Methods

### Study design

TUMIKIA (Tuangamize Minyoo Kenya Imarisha Afya; Swahili for Eradicate Worms in Kenya for Better Health) was a cluster-randomised controlled trial done in Kwale County from March 18, 2015, to May 17, 2017. We originally planned to do the trial in two contrasting settings, but financial and practical considerations meant we prioritised work in Kwale, which had benefited from previous mass drug administration for lymphatic filariasis. The county is mostly rural and environmentally and socioeconomically diverse. Hookworm is the predominant soil-transmitted helminth species. Annual school-based deworming with albendazole has been implemented since 2012,^10^ and community-based treatment for lymphatic filariasis using albendazole and diethylcarbamazine citrate since 2002, albeit intermittently and with low coverage.^11^ Mass treatment is a community-level intervention so we did a cluster-randomised trial; the study objectives pertain to the individual participant level. The unit of randomisation was a community unit, a government health-service delivery structure serving approximately 1000 households. All community units in Kwale county were eligible for enrolment. The rationale and study design have been published previously.^12^

The protocol was approved by the Kenya Medical Research Institute and National Ethics Review Committee (SSC Number 2826) and the London School of Hygiene & Tropical Medicine Ethics Committee (7177). An independent data safety monitoring board monitored the trial and approved the statistical analysis plan. Community meetings were held to explain the nature and purpose of the trial to community members and parents or legal guardians, and written informed consent was obtained for participation in assessment surveys and verbal consent for treatment. No incentives were offered for participation.

### Randomisation and masking

Households were enumerated in consultation with both community health services-led and village-led administration, to provide a baseline sampling frame. This baseline sampling frame was updated after each round of community-wide treatment using household treatment registers. Random assignment of study clusters (1:1:1) to routine school-based treatment, annual community-wide treatment, or biannual community-wide treatment took place after the baseline survey and was stratified by combining subcounty (n=4), hookworm prevalence (below and above 20%, determined in the baseline survey, n=2), and community unit size (below and above a median of 840 households, n=2), leading to 16 strata. Randomisation took place within strata and was done by an independent statistician using computerised random number generation. Owing to the nature of the trial, participants and trial personnel were not masked to allocation, although the identity of the study groups was hidden until the completion of stakeholder engagement and the baseline survey to eliminate participation bias. Community health volunteers and teachers who delivered the treatments were not involved in data collection and laboratory technicians and statisticians were blinded to treatment group.

### Procedures

In total, there were four rounds of treatment in the 2 years. All trial groups received directly observed treatment with albendazole (400 mg) provided from the WHO donation programme to the Government of Kenya through GlaxoSmithKline (London, UK). Trial groups differed by the age range of the target population and the number of treatment rounds in which they were included.

In rounds one (June 4, 2015) and three (May 26, 2016), school-based deworming targeting children aged 2–14 years was provided to all groups as part of the ongoing National School-Based Deworming Programme (NSBDP). On designated deworming days, treatment was offered by trained teachers in primary schools to enrolled and non-enrolled children and younger children in nearby Early Childhood Development Centres. After school-based deworming, the annual community-wide treatment and biannual community-wide treatment groups received community-based (house-to-house) treatment delivered to all individuals aged 2 years and above not treated by the NSBDP. The Kwale County Government coordinated these activities. Treatment was delivered by trained community health volunteers, a cadre of lay health workers selected by their communities to provide basic health services. Each community health volunteer received one day of training on the delivery of community-based treatment and was then responsible for treating approximately 100 households over an 8-day period.

In round two (Nov 23–30, 2015), the national lymphatic filariasis elimination programme used the aforementioned community-based delivery model to target individuals aged 2 years and older with diethylcarbamazine citrate (6 mg/kg) and albendazole in the biannual community-wide treatment group. To preserve the trial design, albendazole was withheld from the school-based deworming and annual community-wide treatment groups, and diethylcarbamazine citrate monotherapy offered. In round four (Oct 28–Nov 4, 2016), only the biannual community-wide treatment group was targeted with diethylcarbamazine citrate and albendazole; no community-based treatment was done in the other two groups because it was delayed until after the final assessment survey. Further details on intervention delivery are provided in the appendix.

Pregnant women and women who had given birth within the past 2 weeks, and those individuals who were seriously ill were ineligible for treatment, due to concerns around the perceived risks of treatment in the study community and absence of clear operation guidance on how to safely reach pregnant women. Passive monitoring of adverse events and severe adverse events was carried out during treatment activities. Before each treatment round, teachers and community health volunteers were trained to recognise and report adverse events and severe adverse events during treatment and follow-up visits, with any severe adverse events arising collated by trial staff, who in turn reported to the data safety monitoring board and Kenya Ministry of Health.

In a change from the published protocol,^12^ cross-sectional community assessment surveys were done after 12 and 24 months; a third assessment survey after 30 months was not possible due to implementation schedules for the national soil-transmitted helminth and lymphatic filariasis control and elimination programmes. The baseline, 12-month and 24-month assessment surveys were done during the 2 months before the NSBDP deworming days (March 18–June 2, 2015, March 20–May 12, 2016, and March 14–May 17, 2017). In each community unit, 225 households were randomly selected from the enumeration listing. Households were excluded if the dwelling could not be found, was vacant, or if no adult resident was home on more than three visits. Household locations were mapped using global positional system coordinates. Household members were enumerated, and a household survey was used to collect data on water and sanitation facilities and proxy indicators of wealth. One household member was randomly selected and asked to provide a stool sample, collected in a plastic pot labelled with a unique identification matrix barcode. Call-backs were arranged either later in the day or early the next day for individuals that could not provide a sample immediately. If the selected individual was unwilling or unable to participate, another household member was randomly selected. Stool samples were transported on the day of collection to a local laboratory, where they were immediately examined microscopically in duplicate using Kato-Katz method, in which slides were examined for hookworm within 1 h of preparation. A senior technician re-examined 10% of slides for quality control.

Treatment coverage was assessed through school and household coverage surveys, done in all schools in the county and all community units in both community-wide treatment groups following rounds 1 and 3, and in all community units in the biannual community-wide treatment group following rounds 2 and 4. All coverage surveys were completed 4–8 weeks after the treatment activities. In schools, class registers were used to randomly select 12 children from each of grades 2, 4, 6, and 8, who were asked their village of residence, if they were present on the deworming day and if they took the tablet. In each community unit, 60 households were randomly selected, and all household members present were asked whether they had been offered deworming treatment by a community health volunteer at home (or by a teacher at school) and whether they had taken the treatment. In both coverage surveys, proxy respondents (other household member, peer or teacher) were accepted when household members or pupils were unable to answer or not present.

All survey and laboratory data generated by study personnel were collected on smartphones and uploaded to a dedicated database maintained on a central server (SurveyCTO, Dobility, Inc, Cambridge, MA); field and laboratory results were linked using the unique identification number barcodes provided on the sample pots.

### Outcomes

The primary outcome was prevalence of hookworm infection among all sampled individuals after 12 and 24 months of intervention. Secondary outcomes were prevalence of *Ascaris lumbricoides* and *Trichuris trichiura*, and intensity of infection for each soil-transmitted helminth species, based on quantitative egg counts, and treatment coverage and costs. An individual was deemed egg positive if one or more eggs were found in any of the slides examined. The intensity of infection was expressed as the number of eggs per gram of stool for each helminth species.

Treatment coverage in household and school coverage surveys was calculated as the proportion of consenting individuals aged 2 years or older (or assenting school-going children) who usually lived in surveyed households (or attended surveyed schools) and who reported being offered treatment that they accepted to swallow, by a community health volunteer at home or by a teacher at school. Coverage was calculated overall by group and by round, and within age strata—2–4 years, 5–14 years, and ≥15 years, adolescent girls (10–19 years), and women of child bearing age (15–49 years). We also calculated total and average cost per person treated per round by community-based (house-to-house) treatment.

### Statistical analysis

Based on a prevalence of 15% at baseline, an intra-cluster correlation coefficient (ICC) of 0·125 and 5% loss to follow-up of community units, a sample size of 40 clusters per group provides 80% power to detect differences between groups of 8% with a significance level of 0·025 to allow for multiple comparisons. The assumed differences between groups lie well within the range predicted by stochastic models of parasite transmission and treatment by alternative strategies.^12^ Prevalence of infection was higher than expected at baseline, the ICC was higher than expected (at 0·238), and variation in cluster size greater than expected, although this did not unduly affect sample size calculations. Treatment coverage surveys were powered at 80% to provide estimates with ±2% precision, assuming a mean coverage of 70% and an ICC of 0·04 in school surveys, and a mean coverage of 50% and an ICC of 0·01 in household surveys. ICC values used in sample size calculations were based on unpublished school and community survey data provided by the NSBDP. Analyses were done on an intention-to-treat basis, excluding individuals without egg count data from at least one slide.

For prevalence of infection, generalised estimating equations were used to estimate the difference between treatment groups after 1 and 2 years of intervention. For infection intensity, zero-inflated negative binomial models were used modelling total numbers of eggs observed, adjusting for inflation using age, sex, and cluster-level prevalence at baseline, and including number of grams of stool examined as an offset (to account for cases in which only one slide was read). For both models, we estimated standard errors and confidence intervals using robust standard errors (the Huber-White Sandwich estimator) to account for correlated outcomes at the level of the community unit. Unadjusted results used only study group as covariates, and adjusted estimates included stratification factors (subcounty, cluster-level prevalence at baseline, and cluster size), rural or urban status, mean cluster-level socioeconomic status, and access to any sanitation and improved water-source at baseline (specified a priori).

Prespecified subgroup analyses were done using formal statistical tests of interaction to compare the consistency (equity) of intervention effects between male and female individuals, socioeconomic strata (defined using a factor analysis of owned assets and household structure performed separately for rural and urban clusters), households with and without reported sanitation access, school-enrolled and non-enrolled children (ages 2–14 years) and adults (aged ≥15 years), and remote (>4 km from a major road) and accessible (≤4 km from a major road) households. Data management and statistical analyses were done using Stata 15.

### Economic analysis

Economic analysis was restricted to community-based treatment approaches, because school-based deworming was not managed by the research team so cost data were not readily available. An ingredients-based approach was used to estimate the cost to the provider of treatment delivered by community health volunteers in rounds 3 and 4 in 2016. Details are provided in the appendix. In brief, both financial costs, which reflect actual monies paid, and economic costs, which reflect the full value of all resources used, were estimated. Costs relating only to research activities were excluded. Data were collected through routine trial reporting and evaluation systems, reviewing financial reports, and key informant interviews. Community health volunteers' time was valued using an opportunity cost approach. All activities were considered recurrent. The value of capital goods was annualised over their useful lifespan using a discount of 3%.^13^ Costs were categorised both by activity and by resource type and estimated for each treatment cluster and group. To compare the costs of community-based delivery alongside or instead of school-based delivery, the within-cluster differences in average costs per person treated were compared between delivery rounds for the biannual group, using a paired *t* test to construct a confidence interval for the cost difference at the 5% level. Univariate sensitivity analysis was done on selected key parameters, including the shadow price of albendazole, daily allowances given to community health volunteers, and discount and exchange rates. Costs are presented in constant 2016 US$ ($1=101·50 Kenyan shillings^14^).

In addition to treatment costs in the trial, we estimated costs for routine programmatic delivery. These were estimated firstly by removing or modifying costs that would not be incurred outside the trial, and secondly, by accounting for economies of scale that would be achieved if the same intervention strategy were extended to all clusters within Kwale County. Specifically, we assumed that the county-level costs, primarily for meetings, would remain fixed, and multiplied the average cost per subcounty and per community unit by the numbers of subcounties and community units in the entire county. Econcomic analyses were done using Microsoft Excel Professional Plus 2016 and RStudio (Version 1.1.453).

This trial is registered with ClinicalTrials.gov, number NCT02397772

### Role of the funding source

The funders of the study had no role in study design, data collection, data analysis, data interpretation, or writing of the report. The corresponding author had full access to all the data in the study and had final responsibility for the decision to submit for publication.

## Results

**Figure 1:**
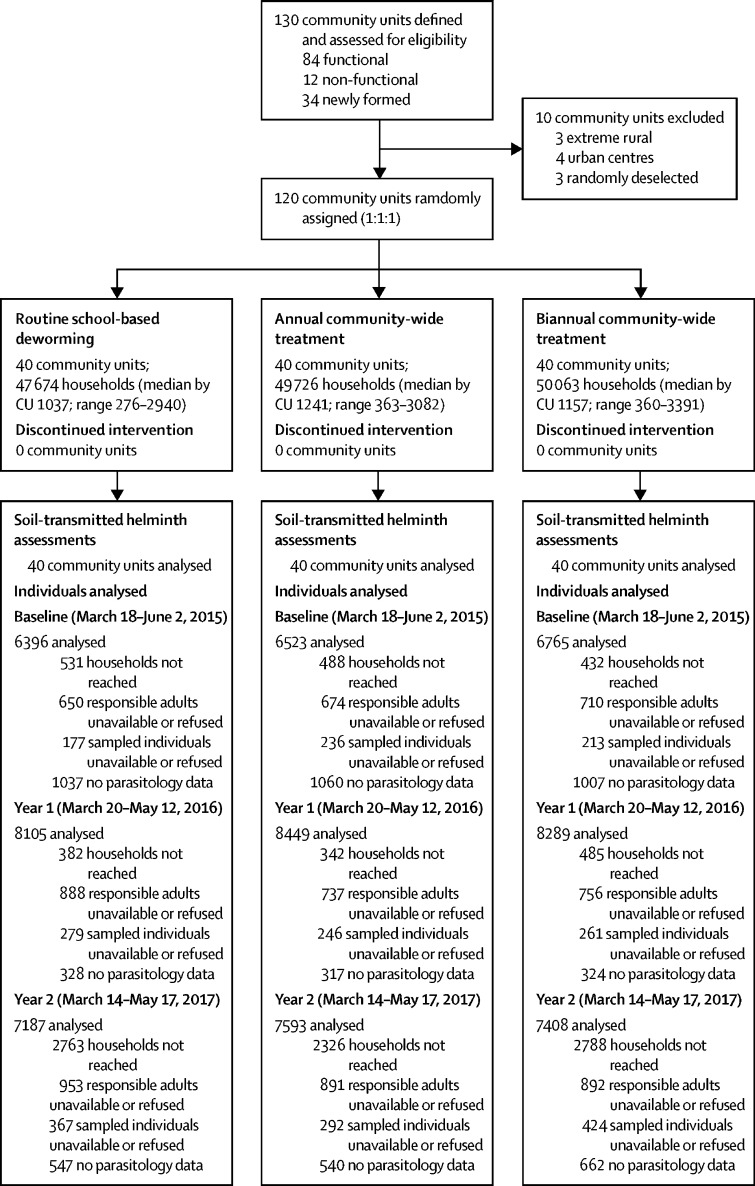
Study profile 120 community units were randomly assigned to either routine school-based deworming or community-wide treatment delivered once or twice a year. No community units were lost to follow up, and none discontinued the intervention. All communities were included in the analysis at 12 and 24 months. Reported cluster sizes are based on the updated sampling frame used for the 24-month evaluation survey. Additional information regarding households and individuals not reached are provided in appendix.

Of the 130 eligible community units, 120 were included in the study and randomly assigned (figure 1), with seven geographically sparse or urban community units excluded and three community units removed through random deselection. No community units were lost to follow-up during the trial. At the final community survey, 147 463 households were listed in the study area (appendix).

The baseline survey included 19 684 individuals with soil-transmitted helminth data (table 1; appendix). Baseline and demographic characteristics were similar across all three groups. Overall prevalence of hookworm was 19·1% (95% CI 16·4–21·7) and prevalence of *T trichiura* was 3·6% (2·6–4·6). *A lumbricoides* was very rare (prevalence <0·5%) and was not analysed further.

**Table 1:** Baseline characteristics of the study population

		**School-based deworming**	**Annual community-wide treatment**	**Biannual community-wide treatment**
**Cluster characteristics (n=120)**				
Total included		40	40	40
Sanitation coverage*		53·5% (2·1%–98·3%)	53·0% (5·3%–92·8%)	49·7% (1·4%–96·2%)
Urban†		4 (10·0%)	2 (5·0%)	3 (7·5%)
Hard to reach‡		3 (7·5%)	4 (10·0%)	4 (10·0%)
Arid§		7 (17·5%)	3 (7·5%)	5 (12·5%)
**Households surveyed (n=23 414)**				
Total included		7610	7819	7985
Number of household members		5 (1–22)	5 (1–23)	5 (1–21)
Asset index score¶		0·47 (−0·01 to 2·21)	0·47 (−0·01 to 2·21)	0·47 (−0·01 to 2·21)
Living in poorest quintile‖		2339 (30·7%)	2407 (30·8%)	2394 (30·0%)
Electricity to household		742 (9·8%)	690 (8·8%)	706 (8·8%)
Owns a bicycle		2315 (30·4%)	2445 (31·3%)	2455 (30·8%)
Earth floor		5797 (76·2%)	6270 (80·2%)	6234 (78·1%)
Household toilet facility access				
	None	3463 (45·5%) of 7605	3546 (45·4%) of 7816	4035 (50·6%) of 7977
	Shared access	1801 (23·7%) of 7605	1907 (24·4%) of 7816	1855 (23·3%) of 7977
	Private access	2341 (30·8%) of 7605	2363 (30·2%) of 7816	2087 (26·2%) of 7977
	Soap and water observed at toilet**	255 (8·2%) of 3095	227 (7·2%) of 3175	207 (6·9%) of 2998
	Toilet facility has washable slab††	1768 (56·1%) of 3150	1534 (47·5%) of 3230	1662 (54·7%) of 3039
Improved water source		3977 (52·6%) of 7556	4589 (58·8%) of 7800	4022 (50·4%) of 7978
Water source <30 min		6138 (81·2%) of 7561	6322 (81·2%) of 7789	6383 (80·4%) of 7940
**Survey participants (n=22 788)**				
Total included		7433	7583	7772
Male/female participants		3017 (40·6%)/4416 (59·4%)	3069 (40·5%)/4514 (59·5%)	3102 (39·9%)/4670 (60·1%)
	<5 years	630 (8·5%)	599 (8·0%)	622 (8·0%)
	5–14 years	2197 (29·6%)	2321 (30·6%)	2378 (30·6%)
	≥15 years	4606 (62·0%)	4663 (61·5%)	4772 (61·4%)
Attending primary school (ages 5–14 yrs)		1980 (90·1%) of 2197	2098 (90·4%) of 2321	2126 (89·4%) of 2378
Observed wearing shoes		3500 (47·2%)	3416 (45·1%)	3406 (43·9%)
Dewormed in past year:		1814 (24·6%)	1905 (25·4%)	1966 (25·6%)
	At school	1204 (66·4%) of 1814	1352 (71·1%) of 1905	1337 (68·0%) of 1966
	At health centre	359 (19·8%) of 1814	358 (19·0%) of 1905	380 (19·3%) of 1966
	Other location or source	251 (13·8%) of 1814	193 (10·1%) of 1905	249 (12·7%) of 1966
**Participants with soil-transmitted helminth data (n=19 684)**				
Total included		6396	6523	6765
Prevalence of soil-transmitted helminth infection				
	Hookworm	1187 (18·6%)	1168 (17·9%)	1396 (20·6%)
	*Ascaris lumbricoides*	30 (0·5%)	18 (0·3%)	30 (0·4%)
	*Trichuris trichiura*	272 (4·3%)	189 (2·9%)	250 (3·7%)
Mean intensity of soil-transmitted helminth infection (eggs per gram)				
	Hookworm	169·7 (1248·2)	175·1 (1965·2)	158·2 (1002·3)
	*Ascaris lumbricoides*	62·6 (2023·3)	18·9 (648·2)	31·0 (808·7)
	*Trichuris trichiura*	12·7 (205·0)	8·3 (145·0)	29·3 (1245·8)

Data are n (%), median (range), or mean (SD). Data were collected during a household-based cross-sectional survey done from March, 2015, to May, 2015.

* Defined as the proportion of households who report having access to a toilet facility.

† Defined as >75% households in areas with population density >1000 persons per km^2^.

‡ Defined as >75% of households <4 km from major road.

§ Defined as >75% households in arid or semi-arid areas.

¶ Based on factor analysis of owned assets and household structure.

‖ From asset index score.

** Data available for 9268 households with toilet and handwashing facilities on premises that agreed to direct observation.

†† Data available for 9419 households with toilet facilities on premises that agreed to direct observation.

**Figure 2:**
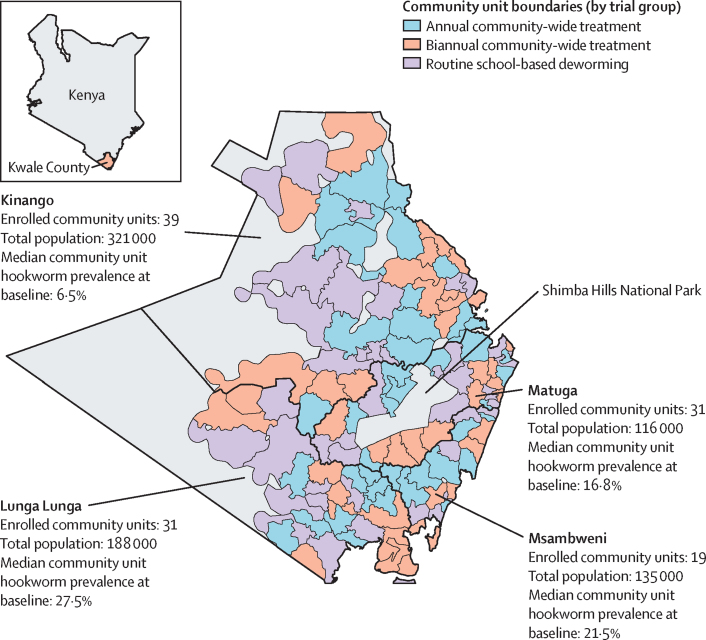
Map of the study area Kwale County consists of four subcounties, all of which were included in the trial. The inset shows the location of Kwale County in Kenya.

All 120 community units received the interventions to which they were randomly assigned (figure 2). Treatment coverage remained relatively consistent across rounds (table 2). Coverage of school-enrolled children aged 5–14 years through school-based deworming was 88·2% (95% CI 87·6–88·7) in May, 2016, and 88·0% (87·5–88·5%) in May, 2017, as reported during school surveys, and did not differ by trial group. Estimates from school surveys were similar to those from household surveys after accounting for school enrolment rates (appendix).

**Table 2:** Reported treatment coverage

			**Annual community-wide treatment arm**		**Biannual community-wide treatment arm**			
			Round 1	Round 3	Round 1	Round 2	Round 3	Round 4
Households reporting community health volunteer visit			2074 (90·8%)	2375 (95·2%)	2074 (91·4%)	2174 (88·3%)	2330 (95·3%)	2112 (92·6%)
Eligible individuals interviewed			12 102	12 227	11 453	11 276	11 908	11 122
Eligible population treatment coverage			9765 (80·7%)	10 140 (82·9%)	9220 (80·5%)	8701 (77·2%)	9882 (83·0%)	8231 (74·0%)
Treatment coverage by age group								
	Pre-school-aged children (2–4 years)							
		Treated (total)	83·1% (80·2–86·0)	77·8 (74·3–81·2)	84·0% (81·3–86·6)	77·3% (72·7–81·8)	79·2% (76·4–82·1)	73·8% (69·5–78·0)
		In school	15·1% (12·9–17·3)	14·5% (12·0–16·9)	15·6% (12·9–18·2)	..	14·5% (12·1–17·0)	..
		At home	68·0% (64·9–72·8)	62·7% (58·8–66·6)	68·4% (65·4–73·5)	..	64·6% (61·6–67·6)	..
	School-aged children (5–14 years)							
		Treated (total)	91·0% (88·9–93·1)	89·5% (87·1–91·8)	90·5% (88·5–92·4)	81·8% (78·5–85·0)	89·6% (87·2–91·9)	79·9% (76·8–83·0)
		In school	68·8% (65·8–71·8)	66·5% (63·3–69·7)	65·8% (62·4–69·2)	..	64·8% (60·2–69·4)	..
		At home	25·1% (22·2–28·1)	21·4% (18·6–84·2)	24·7% (23·8–31·0)	..	23·8% (20·3–27·3)	..
	Adults (≥15 years)							
		Treated (total)	73·7% (72·0–75·4)	79·7% (78·4–81·1)	74·2% (72·3–76·0)	74·5% (71·6–77·3)	79·6% (78·1–81·2)	70·4% (67·7–73·0)
		In school	10·8% (9·8–11·8)	7·7% (6·9–8·6)	9·3% (8·4–10·2)	..	7·7% (6·8–8·5)	..
		At home	63·0% (61·3–65·5)	71·7% (70·0–73·4)	64·9% (63·2–67·3)	..	71·5% (70·0–73·1)	..
	Adolescent girls (10–19 years)							
		Treated (total)	81·1% (78·5–83·7)	84·4% (82·0–86·7)	81·7% (79·2–84·3)	79·0% (75·9–82·0)	85·1% (82·7–87·4)	78·0% (74·6–81·4)
		In school	59·6% (56·0–63·1)	55·2% (52·0–58·4)	57·0% (53·5–60·5)	..	55·8% (51·3–60·4)	..
		At home	21·51% (20·7–26·5)	27·9% (24·8–31·1)	27·1% (24·0–30·2)	..	28·5% (25·4–31·7)	..
	Women of reproductive age (15–49 years)							
		Treated (total)	73·7% (71·7–75·7)	81·6% (80·0–83·2)	74·1% (71·8–76·4)	75·1% (72·3–77·9)	80·4% (78·6–82·2)	73·1% (70·1–76·1)
		In school	11·3% (9·8–12·9)	8·0% (7·0–9·0)	9·7% (8·5–10·9)	..	8·0% (6·8–9·1)	..
		At home	62·4% (60·3–65·2)	73·2% (71·2–75·2)	64·4% (62·8–67·0)	..	72·1% (70·4–73·7)	..

Coverage data are stratified by trial target populations (pre-school-aged children, school-aged children, and adults) and additional WHO-defined target groups (adolescent girls and women of reproductive age).

Coverage of the eligible population in the annual community-wide treatment group was 80·7% (95% CI 79·1–82·2) in 2015 and 82·9% (81·5–84·3) in 2016, and ranged from 74·0% (71·5–76·5) to 83·0% (81·7–84·3%) across the four rounds in the biannual community-wide treatment group (table 2). Community health volunteers reached more than 88% of households in each treatment round, and coverage did not fall below 70% in any of the demographic groups considered.

The 12-month assessment survey included 24 843 individuals, and the 24-month assessment surveys included 22 188 (figure 1; appendix). Characteristics of consenting households and individuals, and for participants with soil-transmitted helminth data who constitute the final analysis datasets, are provided in the appendix. Refusal rates and household and demographic characteristics were similar across all three groups, with adult males under-represented. After 2 years of intervention, the community prevalence of hookworm was 13·8% (95% CI 10·5–17·1) in the school-based deworming group, 8·0% (6·0–10·1) in the annual community-wide treatment group, and 6·2% (4·9–7·5) in the biannual community-wide treatment group (table 3). Compared with school-based deworming, risk of hookworm infection was reduced in the annual (risk ratio 0·59, 95% CI 0·42–0·83) and biannual (0·46, 0·33–0·63) community-wide treatment groups (table 3). Intensity of hookworm infection among all community members was also reduced in the annual community-wide treatment group (intensity rate ratio 0·39, 95% CI 0·27–0·55) and biannual community-wide treatment groups (0·30, 0·19–0·48) relative to those community units receiving school-based deworming, with the greatest reductions observed after 12 months (appendix). Adjusted risk ratio and intensity rate ratio were similar in magnitude and precision to unadjusted estimates. The prevalence or intensity of *T trichiura* did not differ by treatment group (table 3; appendix).

**Table 3:** Effects of annual and biannual community-wide treatment relative to annual school-based deworming on prevalence of hookworm and *Trichuris trichiura*

		**Number positive of total respondents**	**Community prevalence (95% CI)**	**Absolute percentage change from baseline (95% CI)**	**Unadjusted risk ratio (95% CI)**	**p value**	Adjusted risk ratio*(95% CI)	**p value**
**Hookworm**								
12-month assessment								
	School-based deworming	1284 of 7957	16·1% (12·1 to 20·1)	−2·4% (−10·9 to 6·1)	1 (ref)	..	1 (ref)	..
	Annual community-wide treatment	984 of 8355	11·8% (9·0 to 14·6)	−6·1% (−13·6 to −1·3)	0·73 (0·52 to 1·03)	..	0·77 (0·65 to 0·91)	..
	Biannual community-wide treatment	836 of 8177	10·2% (7·6 to 12·9)	−10·4% (−15·5 to −6·0)	0·64 (0·45 to 0·92)	0·04	0·65 (0·53 to 0·78)	<0·001
24-month assessment								
	School-based deworming	972 of 7058	13·8% (10·5 to 17·0)	−4·8% (−13·0 to 3·5)	1 (ref)	..	1 (ref)	..
	Annual community-wide treatment	597 of 7446	8·0% (6·0 to 10·1)	−9·9% (−16·8 to −3·0)	0·59 (0·42 to 0·83)	..	0·64 (0·52 to 0·78)	..
	Biannual community-wide treatment	453 of 7281	6·2% (4·9 to 7·5)	−14·4% (−21·4 to −7·4)	0·46 (0·33 to 0·63)	<0·001	0·48 (0·41 to 0·57)	<0·001
***Trichuris trichiura***								
12-month assessment								
	School-based deworming	296 of 7957	3·7% (1·8 to 5·7)	−0·5% (−6·2 to 5·2)	1 (ref)	..	1 (ref)	..
	Annual community-wide treatment	223 of 8355	2·7% (1·7 to 3·6)	−0·2% (−4·5 to 4·1)	0·70 (0·37 to 1·32)	..	1·18 (0·80 to 1·74)	..
	Biannual community-wide treatment	287 of 8177	3·5% (1·9 to 5·2)	−0·2% (−5·6 to 5·2)	0·90 (0·45 to 1·81)	0·47	1·16 (0·82 to 1·65)	0·63
24-month assessment								
	School-based deworming	292 of 7058	4·1% (1·9 to 6·4)	−0·1% (−5·9 to 5·7)	1 (ref)	..	1 (ref)	..
	Annual community-wide treatment	197 of 7446	2·6% (1·7 to 3·6)	−0·3% (−4·7 to 4·2)	0·65 (0·34 to 1·24)	..	1·20 (0·86 to 1·68)	..
	Biannual community-wide treatment	237 of 7281	3·3% (1·8 to 4·7)	−0·4% (−5·8 to 4·9)	0·80 (0·40 to 1·61)	0·41	1·01 (0·77 to 1·34)	0·43

p values correspond to the treatment group categorical variable.

* Adjusted for stratification factors (subcounty, baseline cluster prevalence, and cluster size), urban or rural status and baseline cluster mean socioeconomic status, access to sanitation, and access to improved water. Sampling was done at randomly selected households, selecting one household member to participate at random.

None of the demographic or socioeconomic characteristics we explored were found to influence the reduction in hookworm infection risk observed in those receiving annual or biannual community-wide treatment relative to school-based treatment. Specifically, we found no evidence of effect differences between male and female individuals, children (both attending and not attending school) and adults, those living in the poorest and least poor households, those with and without access to private sanitation and those living in remote or accessible households (appendix). This finding was consistent for hookworm infection intensity.

In 2016 (treatment rounds three and four of the trial), the economic cost of house-to-house treatment by community health volunteers in the biannual community-wide treatment group ($214 589) was 112% greater than in the annual community-wide treatment group ($101 413). The economic cost of house-to-house delivery was $0·84 per person treated in the annual community-wide treatment group, with wide variation across the county (range by cluster $0·55–$1·56), and $0·76 per person treated per round in the biannual community-wide treatment group (range by cluster $0·49–$1·85; table 4). Within the biannual group, treatment round 4 (which targeted all community members) cost $0·68 per person treated (table 4); across clusters, this was a mean of $0·23 (95% CI 0·16–0·29) less per treatment administered than delivery alongside school-based deworming, which only included those people not treated in school. Most costs (90·5%) were financial (actual monies paid), and the rest were non-financial (eg, community health volunteer opportunity costs). Central administration (33·6%) and drug administration (26·7%) were the most expensive activities, and personnel accounted for 67·5% of costs. Univariate sensitivity analysis of key selected parameters suggested costs were most sensitive to community health volunteer daily allowance. In our routine implementation scenario analysis, we estimated that community-wide treatment of the whole county targeting all ages would have an economic cost of $0·33 per person treated, assuming that coverage levels could be maintained. This projection assumed savings of $0·28 per person treated per round from removing implementation costs that would not be incurred outside of the trial context and further savings of $0·07 from economies of scale. All activities are repeated for each round, so a biannual strategy would be expected to cost approximately twice as much as an annual strategy. Further details of the cost results are included in the appendix.

**Table 4:** Economic cost of community-wide drug administration by trial group and round

	**Summary for all clusters**			**Variation across clusters**	
	Number of treatments administered*	Total cost	Cost per treatment administered	Median number of persons treated (range)	Median cluster cost per person treated (range)
**Annual community-wide treatment**					
Round 3	120 083	$101 413	$0·84	2914 (1303–6075)	$0·86 (0·55–1·56)
**Biannual community-wide treatment**					
Round 3	115 279	$100 454	$0·87	2822 (689–5892)	$0·90 (0·57–2·62)
Round 4	168 130	$114 135	$0·68	4206 (1339–8410)	$0·68 (0·43–1·58)
Rounds 3 and 4	283 409	$214 589	$0·76†	7246 (2121–14 302)	$0·74† (0·49–1·85)

Costs presented in constant 2016 US$.

* Includes only those treated by the community health volunteers and not those treated by teachers at school.

† Mean cost per person treated per round.

During the treatment periods, trial staff were notified of one death attributed to malaria and one hospitalisation attributed to bone formation within the biannual treatment group as well as one hospitalisation due to diethylcarbamazine overdose in a community not included in the trial; medical review determined that none of these related to albendazole delivery (appendix). Non-serious adverse events were difficult to detect in the context of this trial, and none were reported.

## Discussion

Community-wide treatment with albendazole significantly reduced the prevalence and intensity of hookworm among all ages compared with school-based treatment. The effect of community-wide treatment was similar for both annual and biannual treatment, and for different demographic and socioeconomic subgroups. Although our trial was short, these results are in line with the reductions anticipated by mathematical models developed before the trial,^12^ and highlight the potential of community-wide treatment targeting all ages to reduce infection prevalence and potentially interrupt transmission of hookworm. The results are also consistent with a meta-analysis that found that community-wide treatment was more effective than school-based treatment in reducing hookworm infection among school-aged children,^6^ and provide additional data on the effects among other age groups. An ongoing set of trials is investigating whether these reductions in infection can be translated into the interruption of transmission, once treatment has stopped and following a period of surveillance.^15^

Community-wide neglected tropical disease treatment programmes aim to achieve sustained, universal coverage. An acknowledged risk of universal coverage goals is that, without additional targeted effort, programmes might not reach the most disadvantaged groups. These groups will often be at increased risk of infection, which might lead to a rise in inequality.^16^ Although community health worker programmes have been argued to help health systems reach the poorest and hardest to access, remarkably few studies have assessed the equity of community health worker programmes adopting universal approaches, with nearly all those available focusing on family health, maternal and child health, and malaria control.^17^ Given that community-based programmes, including neglected tropical disease treatment, are increasingly advocated as a means to ensure and monitor universal health coverage,^9^ robust evidence of equity is crucial. The observation of high treatment coverage and equitable effects across all demographic and socioeconomic groups shown here is encouraging and suggests the use of community health systems facilitated equitable delivery of the intervention. Future work should continue to evaluate the equity of neglected tropical disease treatment delivery platforms for access and health effects.

Several factors contributed to the high uptake of treatment. The intervention was delivered through a partnership model closely coordinated by the county government, but with substantial administrative and logistical support and training provided by trial personnel. This collaboration ensured a reliable distribution cascade for resources and drugs, while maintaining high local stakeholder and community ownership, and contributed to ongoing human resource and data capacity within the local health system. Community health volunteers were remunerated for their work, with a degree of performance-based pay. Communication had a large role in the success of the programme, with community mobilisation done through meetings held with high-level county officers down to village elders and review meetings after implementation to ensure that challenges in previous rounds were addressed. A systematic review^18^ of factors that shape implementation of mass drug administration for lymphatic filariasis in sub-Saharan Africa suggested that programmes are particularly vulnerable to several elements that were carefully managed in our setting, including geographical demarcation of catchment areas, timing of drug deliveries, number of drug distributors and allocated households, and appropriate incentives and training for distributors. Therefore, caution about whether similar coverage can be achieved during routine programmatic activities is necessary.

Our high level of investment in implementation is reflected in the relatively high estimated economic cost of treating the whole community in our trial context ($0·68 per person treated per round). Costs per person treated in our trial were higher still when community health volunteers delivered treatment immediately after school-based deworming, because the substantial decrease in the number of people targeted for treatment only led to a small decrease in the total cost of delivery. This finding calls into question the efficiency and usefulness of hybrid delivery platforms that maintain both school-based and community-based delivery of albendazole. Few high quality costing studies of routine delivery of neglected tropical disease interventions exist, and future work in this area is needed, but our cost estimates of soil-transmitted helminth treatment are consistent with those reported by other studies.^19^ For example, Evidence Action (which supports implementation of school-based deworming in Kenya) reported that the total financial cost of the Kenya NSBDP was $0·56 per child treated in the 2014–15 school year.^20^ Furthermore, our scale-up scenario suggests that if coverage levels can be maintained during routine programmatic delivery—a somewhat weighty assumption—the cost per person treated could be as low as $0·33. Further economic analyses are underway.

In 2017, the WHO expanded its strategy for control of soil-transmitted helminth-associated morbidity to also include periodic treatment of adolescent girls, women of reproductive age, and pregnant women and girls after their first trimester.^2^ However, the necessary operational guidance on how to reach these groups needs to be developed and provided to national programmes.^21^ Our results demonstrate that community-based treatment targeting all ages reached substantially more adolescent girls and women of reproductive age than school-based deworming in this setting. This suggests that community-based treatment might provide an appropriate delivery mechanism as countries look to achieve long-term morbidity control goals in all target groups. Although scaling-up existing deworming programmes to target a broader age range increases drug and financial needs in the shorter term, savings in the longer term are probable if transmission can be interrupted and regular treatment is no longer required.

The observed absence of overall difference in effects between biannual and annual community-wide treatment might reflect the slow reinfection rate for hookworm.^22^ By contrast, biannual treatment might prove more effective against *T trichiura* or *A lumbricoides,* which are both characterised by reinfection rates of less than 6 months.^22^ Alternatively, the absence of difference might indicate that the same individuals are receiving treatment in each round, with a core group remaining untreated, thus reducing any benefits provided by a second treatment round.^23^ This observation is further compounded by the substantially increased annual cost of delivering treatment using community health volunteers twice a year when compared with delivering treatment once a year in this low infection intensity setting. Longer-term follow up is warranted to determine whether declines in prevalence become more divergent with subsequent rounds of treatment.

Albendazole is known to have low efficacy against *T trichiura,* which might partially explain the absence of treatment effect seen here. Evidence highlights the benefit of combination therapy with albendazole plus ivermectin for *T trichiura*,^24^ with ivermectin now indicated for soil-transmitted helminths on the WHO List of Essential Medicines.^25^ The observed low prevalence and focal distribution of *T trichiura* and *A lumbricoides,* and consequent low statistical power, are also likely explanations for absence of differential effect by treatment strategy. By contrast, a non-randomised community trial of biannual mass treatment with albendazole done in Republic of Congo^26^ decreased the prevalence of *A lumbricoides* from 56·5% at baseline to 12·9% after 3 years, *T trichiura* from 78·4% to 59·4%, and hookworm from 6·5% to 0%.

This trial focused exclusively on the delivery of treatment and did not address the adoption of sanitation hardware and hygiene behaviours that are important in reducing environmental contamination and exposure to soil-transmitted helminth infectious stages. Although inadequate water, sanitation, and hygiene is a recognised risk factor for soil-transmitted helminth infection,^27^ there is less evidence of an effect of water, sanitation, and hygiene interventions on soil-transmitted helminth prevalence or intensity.^28^, ^29^ This scarcity of evidence exists in part because these interventions are challenging to implement and measure at scale; they are also very costly relative to mass drug administration. Although access to sanitation was very heterogeneous across our site, the relative effects of the interventions were the same for those with access to a toilet and those without, suggesting that community-wide treatment was effective even in heavily contaminated environments.

A rational concern for programmes that rely on mass drug administration is the emergence of drug resistance. Experience of anthelmintic resistance in livestock suggests that increasing the population treatment coverage (and hence reducing parasite refugia) might increase selection pressure, but the empirical evidence for this is mixed and we do not have conclusive evidence of resistance to albendazole in human soil-transmitted helminths, despite long-standing lymphatic filariasis programmes that provide community-wide albendazole. However, programmes need to routinely monitor drug efficacy and potential drug resistance.^30^

The trial had several limitations. First, due to logistical considerations, we were unable to do a full census before the start of the trial, and instead relied upon household lists generated in consultation with health and village administration, which were updated on a biannual basis. This reliance is reflected in the substantial increase in the final sampling frame, from 101 071 households at baseline to 147 463 at the 24-month assessment survey. However, no major differences occurred in household or individual characteristics between the baseline and 24-month assessment surveys. However, this strategy might have led to us continually missing the most marginalised households, which might well be those at greatest risk of infection and the least likely to be included within community-wide campaigns. Second, although the sampling strategy used for the parasitological surveys was age-representative, adults, especially men, were least likely to consent to take part and provide a sample. Third, owing to implementation schedules for the national soil-transmitted helminth and lymphatic filariasis control and elimination programmes, the duration since last treatment differed between groups; the biannual community-wide treatment group were treated 6 months later than the annual community-wide and routine school-based deworming groups. This difference will have exaggerated any differences in infection between annual and biannual strategies. Fourth, although findings from coverage surveys after treatment were encouraging, we found recruitment for the coverage survey difficult, with approximately 20% of sampled households unavailable during data collection. Therefore, included households might not be representative of the total population at risk, and instead might represent easier-to-reach households more likely to be captured during mass drug administration. Lastly, we relied upon dual Kato-Katz smears for detection of infection. Although this is the primary diagnostic tool recommended by the WHO, it is relatively insensitive especially at low intensities and as such we will have underestimated the true numbers infected.^31^ Newer techniques, such as quantitative PCR, have greater sensitivity than Kato-Katz (90% *vs* 70% sensitivity^32^) and are becoming increasingly important as infection drops to very low levels in some regions of the world.^33^ However, the lower sensitivity of Kato-Katz is unlikely to change the overall conclusion of the study, due to successful randomisation of clusters.

Since 2012, there has been remarkable scale up of geographically targeted school-based deworming programmes, aimed at reducing the morbidity attributable to soil-transmitted helminths in children. Expansion of treatment to all ages might support sustainable interruption of parasite transmission, and thus avoid the need for ongoing mass treatment. The results of this study indicate that expansion of treatment to adults using community health volunteers represents a feasible strategy to reduce hookworm infections in the community. The findings also highlight the equity of the neglected tropical disease delivery platform, its ability to reach the poorest, most marginalised communities, and its potential to deliver other health interventions and, thus, contribute towards universal health-care coverage.

## Data sharing statement

Data collected from this study, including de-identified individual participant data, will be made available upon publication to members of the scientific and medical community for non-commercial use only, upon email request to the corresponding author. Data will be stored in Data Compass, the London School of Hygiene & Tropical Medicine digital data repository.
