# Supplementary material for: Effects, equity, and cost of school-based and community-wide treatment strategies for soil-transmitted helminths in Kenya: a cluster-randomised controlled trial
Source: Lancet. 2019 May 18;393(10185):2039–50. doi: 10.1016/S0140-6736(18)32591-1 (PMC6525786; doi:10.1016/S0140-6736(18)32591-1)
Supplement: Supplementary appendix [file mmc1.pdf]

APPENDIX
(Impact, equity and cost of school-based and community-wide treatment strategies for soil-transmitted helminths in Kenya: a cluster-randomised controlled trial)

TABLE OF CONTENTS

Web appendix 1: CONSORT 2010 Checklist with Extensions for Cluster Randomised Trials 2

Web Appendix 2: Delivery of trial interventions during the TUMIKIA trial: partnership with national NTD control and elimination programmes. 5

Web Figure 1: Timeline schematic for major trial activities by arm. 7

Web Figure 2: Consort Diagram for Baseline Survey 8

Web Figure 3: Consort Diagram for 12 month assessment survey (after one year of intervention) 9

Web Figure 4: Consort Diagram for 24 month assessment survey (after two years of intervention) 10

Web Table 2: Characteristics of the study population included in the 12-month assessment survey. 11

Web Table 3: Characteristics of the study population included in the 24-month assessment survey. 12

Web Appendix 3: Additional results (secondary outcomes) 13

Web Table 4 Effect of annual and biannual community-wide treatment relative to school-based deworming on intensity (as measured by eggs per gram (EPG)) of hookworm and *T. trichiura* after one and two years of intervention. 13

Web Table 5 Effect of annual and biannual community-wide treatment relative to annual school-based deworming on prevalence of hookworm after twenty-four months of intervention in key sub-groups 14

Web Table 6 Effect of annual and biannual community-wide treatment relative to annual school-based deworming on hookworm infection intensity after twenty-four months of intervention in key sub-groups 15

Web Table 7: Treatment coverage during school-based deworming, as measured during treatment coverage surveys conducted in schools. 16

Web Appendix 3: Safety Report across all four rounds of implementation. 17

Web Table 8: Severe adverse events (SAEs) reported to trial data & safety monitoring board between April 2015 and May 2017 17

Web Appendix 4: Details of Intervention Costing 18

Web Table 9: Cost matrix describing breakdown of costs included within each of the cost categories, highlighting implementation costs specific to the trial context that are removed for the implementation scenario. 20

Web Figure 5: Cost of albendazole distribution immediately following a school-based strategy (round 3) by resource type and activity 22

Web Figure 6: Heterogeneity of cost per person treated by community-wide treatment by trial arm and round, with variation by cluster. 23

Web Table 10: Financial cost of community-based drug administration by trial arm and round. 24

Web Table 11: Economic cost of community-based drug administration by trial arm and round. 25

Web Table 12: Economic and financial cost of routine intervention scenario for community-wide treatment. 26

Web Appendix 6: Data Sharing Statement 27

Web Appendix References 28

THE LANCET

Supplementary appendix

This appendix formed part of the original submission and has been peer reviewed. We post it as supplied by the authors.

Supplement to: Pullan RL, Halliday KE, Oswald WE, et al. Effects, equity, and cost of school-based and community-wide treatment strategies for soil-transmitted helminths in Kenya: a cluster-randomised controlled trial. *Lancet* 2019; published online April 18. http://dx.doi.org/10.1016/S0140-6736(18)32591-1.

Web appendix 1: CONSORT 2010 Checklist with Extensions for Cluster Randomised Trials

SECTION/TOPIC AND DESCRIPTION		RESPONSE AND LOCATION
**TITLE AND ABSTRACT**		
1a	Identification as a randomised trial in the title; Identification as a cluster randomised trial in the title	Stated explicitly in **title**.
1b	See linked abstract table below	
**INTRODUCTION**		
2a	Scientific background and explanation of rationale; rationale for using a cluster design	Study rationale is described in the introduction; additional rationale for using a cluster design is provided in the **methods** (under **study design**) “Given that mass treatment represents a community-level intervention, a cluster design was used.” We also provide a link to the protocol paper, which presents this in further details.
2b	Specific objectives or hypotheses; whether objectives pertain to the cluster level, the individual participant level or both	We state the overall study objective in the final paragraph of the introduction; we include a clarifying statement in the **methods** (under **study design**) “the study objectives however pertain to the cluster level and individual participant level”. We also provide a link to the protocol paper, which presents this in further details.
**METHODS**		
**Trial Design**		
3a	Description of trial design (such as parallel, factorial) including allocation ratio; definition of cluster and description of how the design features apply to the clusters	Included in **methods** (under **study design**, **randomisation and masking**, **interventions**). Specific details of the cluster definitions and how the trial was applied to clusters are provided under **study design** and **interventions**.
3b	Important changes to methods after trial commencement (such as eligibility criteria), with reasons	Not applicable
**Participants**		
4a	Eligibility criteria for participants; eligibility criteria for clusters	Eligibility criteria for participants are provided in the **methods** (**assessments**); cluster eligibility criteria are provided under **study design**.
4b	Settings and locations where the data were collected	Provided in **methods**, under **interventions** and **assessments**. To provide further context, a map of the study area and clusters is provided in **Figure 2**.
**Interventions**		
5	The interventions for each group with sufficient details to allow replication, including how and when they were actually administered; Whether interventions pertain to the cluster level, the individual participant level or both	Details of the interventions – and the level at which these pertain - are provided in **methods** (under **interventions**).
**Outcomes**		
6a	Completely defined pre-specified primary and secondary outcome measures, including how and when they were assessed; whether outcome measures pertain to the cluster level, the individual participant level or both	All primary and secondary outcome measures are defined in the **methods** (under **outcomes**) including the level at which these pertain; details of how and when they were assessed are included under **assessments**.
6b	Any changes to trial outcomes after the trial commenced, with reasons	Not applicable
**Sample size**		
7a	How sample size was determined; Method of calculation, number of clusters(s) (and whether equal or unequal cluster sizes are assumed), cluster size, a coefficient of intracluster correlation (ICC or *k*), and an indication of its uncertainty	Sample size determination is presented in **methods** (under **statistical analysis**) although we acknowledge that this is a summary. Further details are provided in the protocol paper, for which we provide a link.
7b	When applicable, explanation of any interim analyses and stopping guidelines	Not applicable
**RANDOMISATION**		
**Sequence generation**		
8a	Method used to generate the random allocation sequence;	Included in **methods** (under **randomisation and masking**)
8b	Type of randomisation; details of any restriction (such as blocking and block size)	Included in **methods** (under **randomisation and masking**)
**Allocation concealment mechanism**		
9	Mechanism used to implement the random allocation sequence (such as sequentially numbered containers), describing any steps taken to conceal the sequence until interventions were assigned; Specification that allocation was based on clusters rather than individuals and whether allocation concealment (if any) was at the cluster level, the individual participant level or both	Included in **methods** (under **randomisation and masking**). Additional clarity on allocation blinding at the cluster and individual level is also provided in this section.
**Implementation**		
10a	**Who generated the random allocation sequence, who enrolled clusters, and who assigned clusters to interventions**	Included in **methods** (under **randomisation and masking**).

10b	**Mechanism by which individual participants were included in clusters for the purposes of the trial (such as complete enumeration, random sampling)**	Included in **methods** (under **evaluation**)
10c	From whom consent was sought (representatives of the cluster, or individual cluster members, or both), and whether consent was sought before or after randomisation	Consenting procedures for the intervention is described in the **methods** section (under **study design**), whilst that for the evaluation is included under the **evaluation** section.
**Blinding**		
11a	If done, who was blinded after assignment to interventions (for example, participants, care providers, those assessing outcomes) and how.	Included in **methods** (under **randomisation and masking**).
11b	If relevant, description of the similarity of interventions	A statement explicitly stating the similarities and differences between arms is provided in the **methods** (under **implementation**).
**Statistical methods**		
12a	Statistical methods used to compare groups for primary and secondary outcomes; How clustering was taken into account	Included in **methods** (under **statistical analysis**).
12b	Methods for additional analyses, such as subgroup analyses and adjusted analyses	
**RESULTS**		
**Participant flow (a diagram is strongly recommended)**		
13a	For each group, the numbers of participants who were randomly assigned, received intended treatment, and were analysed for the primary outcome; For each group, the numbers of clusters that were randomly assigned, received intended treatment, and were analysed for the primary outcome	Summary information is provided in the first paragraph of the **results** section. A consolidated flow diagram is provided in **Figure 1.** This includes for each group, the number of households assigned to receive the interventions (without a comprehensive census, it is not possible to state with confidence the total number of individuals assigned) and the numbers of individuals recruited and analysed for the primary outcome in the evaluation surveys.
13b	For each group, losses and exclusions after randomisation, together with reasons; For each group, losses and exclusions for both clusters and individual cluster members	Summary information is provided in the first paragraph of the **results** section, at cluster and individual level. Additional detailed flow diagrams for each cross-section survey provide further information on losses and exclusions.
**Recruitment**		
14a	Dates defining the periods of recruitment and follow-up	These are provided in the **results** section (paragraph 5).
14b	Why the trial ended or was stopped	Not applicable.
**Baseline data**		
15	A table showing baseline demographic and clinical characteristics for each group; Baseline characteristics for the individual and cluster levels as applicable for each group	These details are provided in **Table 2**. This provides baseline characteristics at cluster, household and individual level.
**Numbers analysed**		
16	For each group, number of participants (denominator) included in each analysis and whether the analysis was by original assigned groups; For each group, number of clusters included in each analysis	This information is provided in Table 3 for the primary outcomes, and Tables 2 and 4 for secondary outcomes. We include a statement in the **methods** (under **statistical analysis**) to clarify that all analysis was carried out on an intention-to-treat basis only; all clusters were included in all analyses as described in **Figure 1**.
**Outcomes and estimation**		
17a	For each primary and secondary outcome, results for each group, and the estimated effect size and its precision (such as 95% confidence interval); Results at the individual or cluster level as applicable and a coefficient of intracluster correlation (ICC or k) for each primary outcome	Primary and secondary outcome results are provided in the **results** body text (primary outcome: results, paragraph 5), and in **Tables 2-5**.
17b	For binary outcomes, presentation of both absolute and relative effect sizes is recommended	For the primary outcome, absolute and relative effect sizes are provided in **Table 3.**
**Ancillary analyses**		
18	Results of any other analyses performed, including subgroup analyses and adjusted analyses, distinguishing pre-specified from exploratory	All sub-group and adjusted analysis results are provided in **tables 2-5**. We include a statement that all sub-group analyses were prespecified.
**Harms**		
19	All important harms or unintended effects in each group (for specific guidance see CONSORT for harms)	Severe adverse events are reported in the final paragraph of the **results**. We also acknowledge that "Non-serious adverse events were difficult to detect in the context of this trial, and none were reported."
**DISCUSSION**		
**Limitations**		
20	Trial limitations, addressing sources of potential bias, imprecision, and, if relevant, multiplicity of analyses	Limitations and source of bias are discussed in paragraph 8 of the **discussion**.
**Generalisability**		
21	Generalisability (external validity, applicability) of the trial findings; Generalisability to clusters and/or individual participants (as relevant)	Generalisability is covered in several sections of the **discussion**, including paragraph 3.
**Interpretation**		
22	Interpretation consistent with results, balancing benefits and harms, and considering other relevant evidence;	Provided in the **discussion**.
**OTHER INFORMATION**		
**Registration**		

23	Registration number and name of trial registry	Trial registration number and registry is provided in the **abstract**.
**Protocol**		
24	Where the full trial protocol can be accessed, if available	A reference to the full trial protocol is provided in the **methods** (under **study design**).
**Funding**		
25	Sources of funding and other support (such as supply of drugs), role of funders	A **funding statement** is included.

**Extension of CONSORT for abstracts to report of cluster randomised trials**		
**Title and trial design**	Identification of study as cluster randomised; description of the trial design (e.g. parallel, cluster, non-inferiority)	Explicitly stated in title.
**Methods**		
Participants	Eligibility criteria for participants and the settings where the data were collected; eligibility criteria for clusters	Although not stated explicitly in the abstract, these details are provided in the manuscript body text.
Interventions	Interventions intended for each group	Provided in the abstract methods statement.
Objective	Specific objective or hypothesis; Whether objective or hypothesis pertains to the cluster level, the individual participant level or both	Specific objective provided in abstract background.
Outcome	Clearly defined primary outcome for this report; whether the primary outcome pertains to the cluster level, the individual participant level or both	Primary and secondary outcomes stated in the abstracted methods.
Randomisation	How participants were allocated to interventions; How clusters were allocated to interventions	Although though not stated explicitly in the abstract, these details are provided in the manuscript body text.
Blinding (masking)	Whether or not participants, care givers, and those assessing the outcomes were blinded to group assignment	Although though not stated explicitly in the abstract, these details are provided in the manuscript body text.
**Results**		
Numbers randomised	Number of participants randomized to each group; Number of clusters randomized to each group	Approximate total population size and number of clusters per arm provided in abstract methods statement.
Numbers analysed	Number of participants analysed in each group; Number of clusters analysed in each group	Although though not stated explicitly in the abstract, these details are provided in the manuscript body text.
Outcome	For the primary outcome, a result for each group and the estimated effect size and its precision; Results at the cluster or individual participant level as applicable for each primary outcome estimated effect size and its precision;	Results for primary outcome in each arm stated in the abstract findings statement.
Harms	Important adverse events or side effects	Not applicable.
**Conclusion**	General interpretation of the results	An interpretation statement is provided in the abstract
**Trial registration**	Registration number and name of trial register	Stated in the abstract methods statement.
**Funding**	Source of funding	A funding statement is provided.

Web Appendix 2: Delivery of trial interventions during the TUMIKIA trial: partnership with national NTD control and elimination programmes.

This trial was successfully implemented within the context of two national control programmes: the Kenya National School-based Deworming Programme (NSBDP) and the National Programme to Eliminate LF (NPELF). In total, there were four treatment rounds. The first and third were conducted in partnership with the NSBDP, and the second and fourth in partnership with the NPELF. Here we provide summary details on the delivery of each treatment round. The accompanying schematic presents all trial activities and interventions in a timeline.

Treatment Rounds 1 and 3: partnership with the NSBDP

The Ministry of Education, Science and Technology (MoEST) and the Ministry of Health (MoH) implement the NSBDP, with technical and coordination support provided by Evidence Action, a non-governmental organisation. The TUMIKIA trial was implemented alongside the NSBDP in Kwale, and no adaptations were made to school-based delivery in any study arm. The NSBDP targets both school-aged children (5-14 years) and pre-school aged children (2-4 years). On designated deworming days, primary school teachers (including those in governmental and non-governmental schools) deliver treatment with 400mg albendazole to children aged 2-14 years. Treatment is offered to enrolled children, to children over two years old from nearby Early Childhood Development (ECD) centres, and to non-enrolled school-aged children.

The unit of randomisation for the trial was a community unit (CU), which represents the lowest level of the health system. These do not align with school catchment boundaries (as one school may serve several CUs, and conversely one CU may contain several schools). School-based deworming (SBD) was thus implemented county-wide for the duration of the trial, with CUs randomised to the community-wide treatment (CWT) arms receiving supplementary community-based treatment after the school deworming day. This consisted of community-based (house-to-house) treatment delivered to all individuals aged 2 years and above not treated by the NSBDP. The Kwale County government coordinated these activities. Treatment was delivered by trained community health volunteers (CHVs), a cadre of lay health workers selected by their communities to provide basic health services. Each CHV received a one-day training on the delivery of community-based treatment, and was then responsible for treating approximately 100 households over an eight-day period. In each round, trial personnel provided training and technical support to CHV supervisors and health services administration; no trial personnel were involved in the delivery of treatment to household members.

In 2015 (Round 1) there was a delay of five weeks between SBD and community-based delivery, as treatment could not be delivered during Ramadan; in 2016 (Round 3) community-based treatment immediately followed school-based treatment.

Treatment Rounds 2 and 4: partnership with the NPELF

In Kenya, lymphatic filariasis (LF) is endemic in the coastal regions, including Kwale county. Given that Kenya is non-endemic for onchocerciasis, the recommended antifilarial treatment for mass drug administration (MDA) is single-dose annual mass treatment with diethylcarbamazine citrate (DEC, 6 mg/kg) plus albendazole (400 mg). The Kenyan MoH launched its LF elimination programme in 2002, and due to financial and logistical challenges, has conducted MDA on only an intermittent basis. When the TUMIKIA trial was initiated in March 2015, there was no MDA scheduled for 2015 or 2016.

An LF sentinel site survey conducted in October 2015 suggested ongoing transmission in Kwale county, with LF microfilariae prevalence between 0 and 0.3% across three sentinel site villages.[1] This prompted a renewed commitment to re-start the LF elimination programme in Kenya with support from the WHO-AFRO Regional Office and other partners. Consequently, LF MDA campaigns were conducted in Kwale in November 2015 and November 2016. The Kwale county MoH coordinated activities for these campaigns in partnership with trial personnel and Evidence Action, and they constituted Rounds 2 and 4 of the trial.

To preserve the TUMIKIA design and prevent contamination, the MoH determined that in 2015 (Round 2) albendazole would be withheld from CUs in Kwale randomised to receive SBD or annual CWT, such that they only received DEC-

monotherapy during the LF campaign. Although not the optimal strategy, this was justified based on demonstrated efficacy of DEC mono-treatment in low-endemicity settings.[2-4] CUs in the biannual CWT arm were still targeted for MDA with albendazole-DEC combined therapy. As before, treatment was offered house-to-house by CHVs using the same community-based delivery model. Similarly, whilst trial personnel provided training and technical support, and oversaw the distribution of drugs as far as the study clusters, no trial personnel were involved in the delivery of treatment to household members.

In 2016 (Round 4), implementation was modified and all treatment was withheld from the SBD and annual CWT arms. Community-based treatment with albendazole and DEC was still delivered to the biannual CWT arm. Immediately after the end of the trial, in May 2017, the NPELF delivered MDA with albendazole-DEC combined therapy to all communities in Kwale, again with the support of the TUMIKIA study.

Web Figure 1: Timeline schematic for major trial activities by arm.
Includes information on the timing and duration of baseline and assessment surveys, school-based and community-based treatment, and coverage surveys.

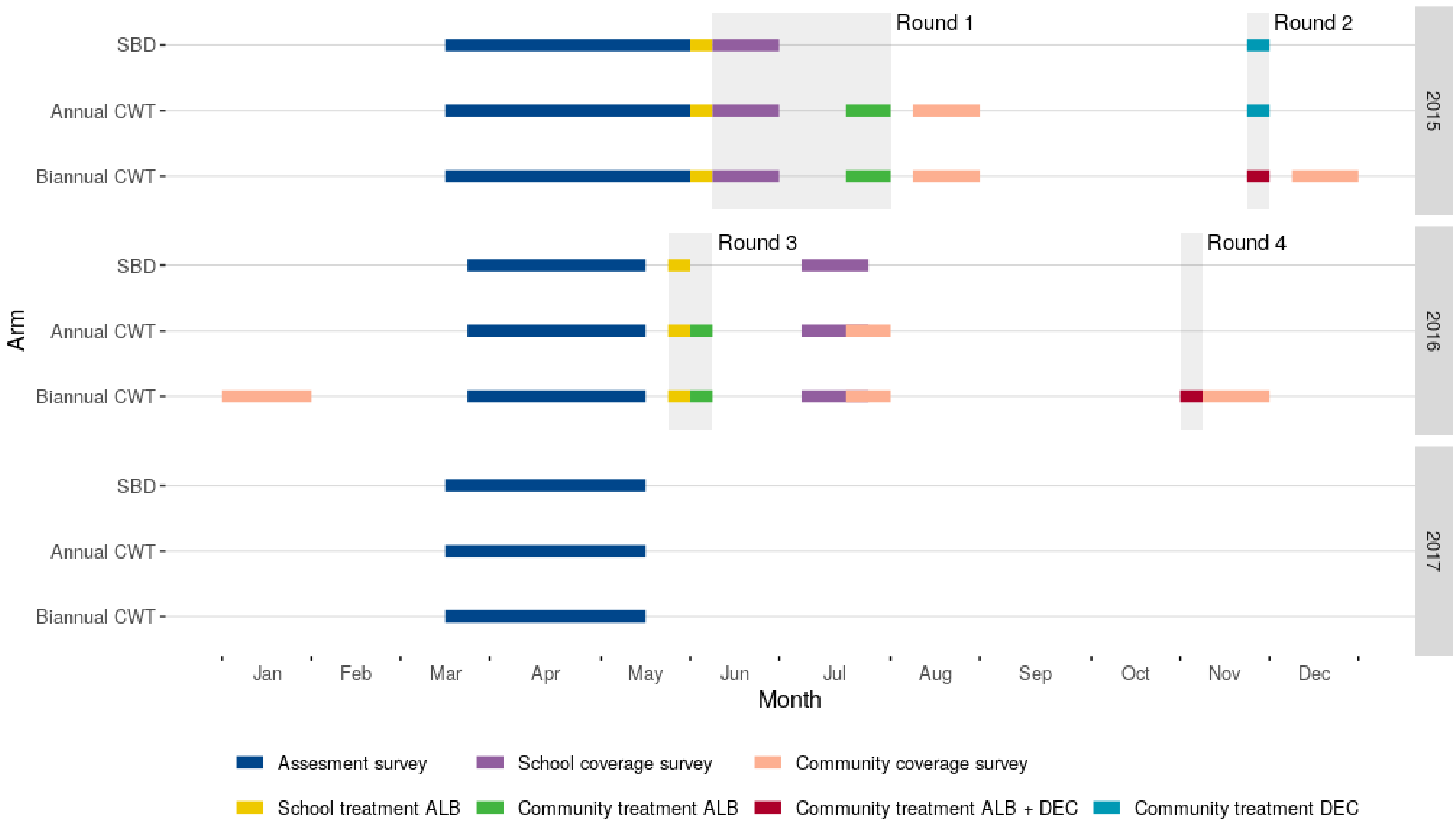

Web Figure 2: Consort Diagram for Baseline Survey

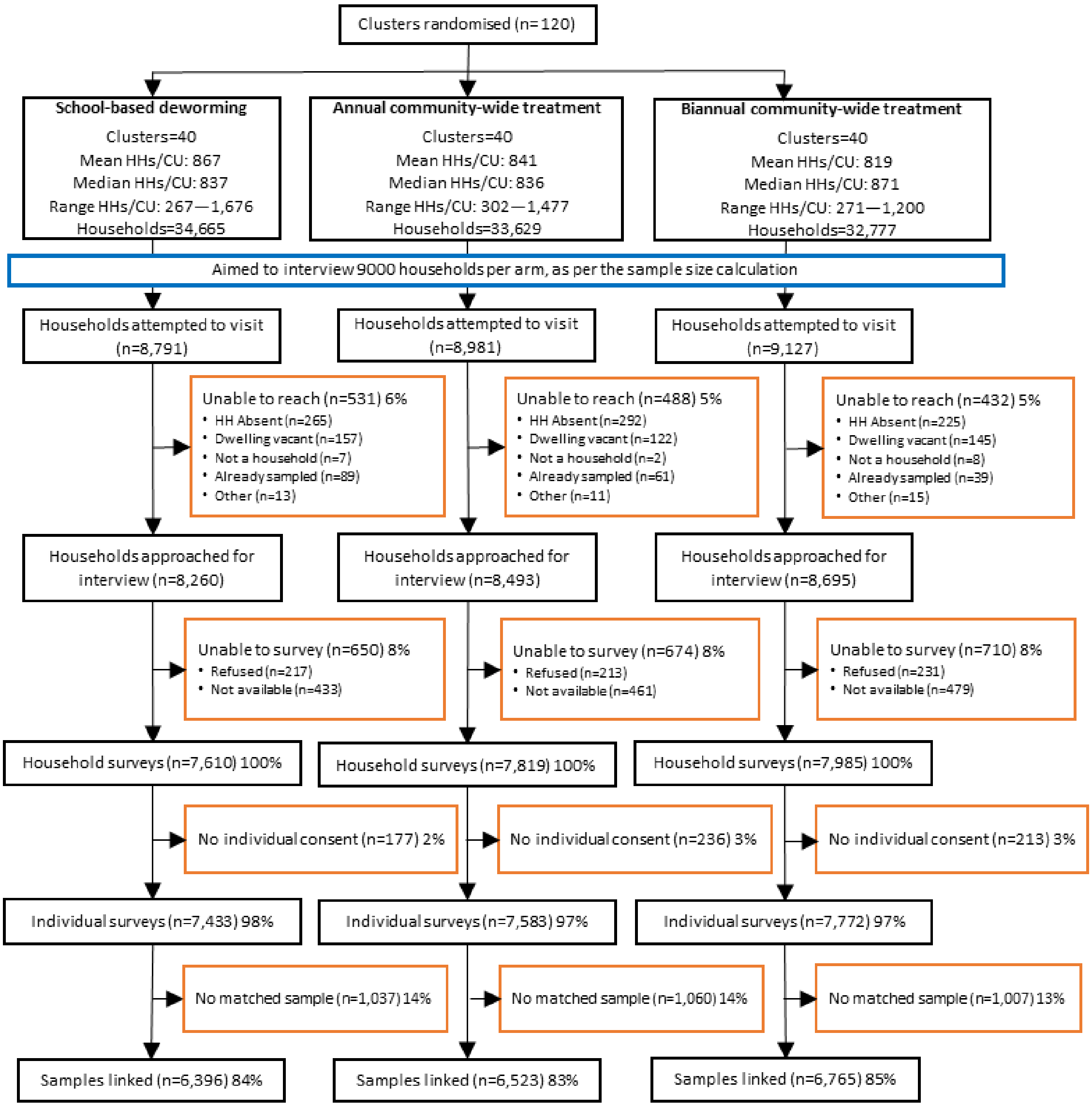

HH; household. CU; community unit

Web Figure 3: Consort Diagram for 12 month assessment survey (after one year of intervention)

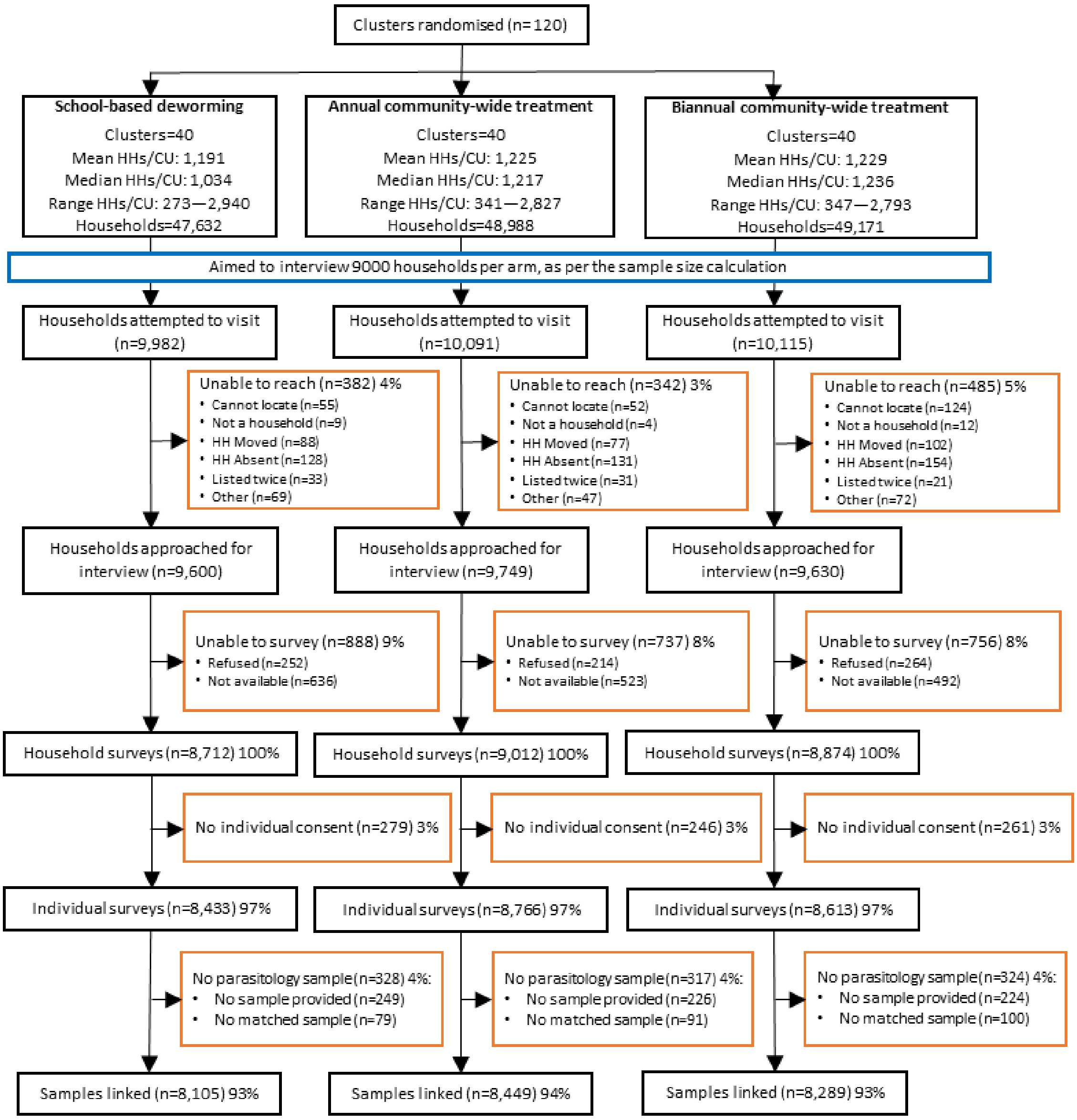

HH; household. CU; community unit

Web Figure 4: Consort Diagram for 24 month assessment survey (after two years of intervention)

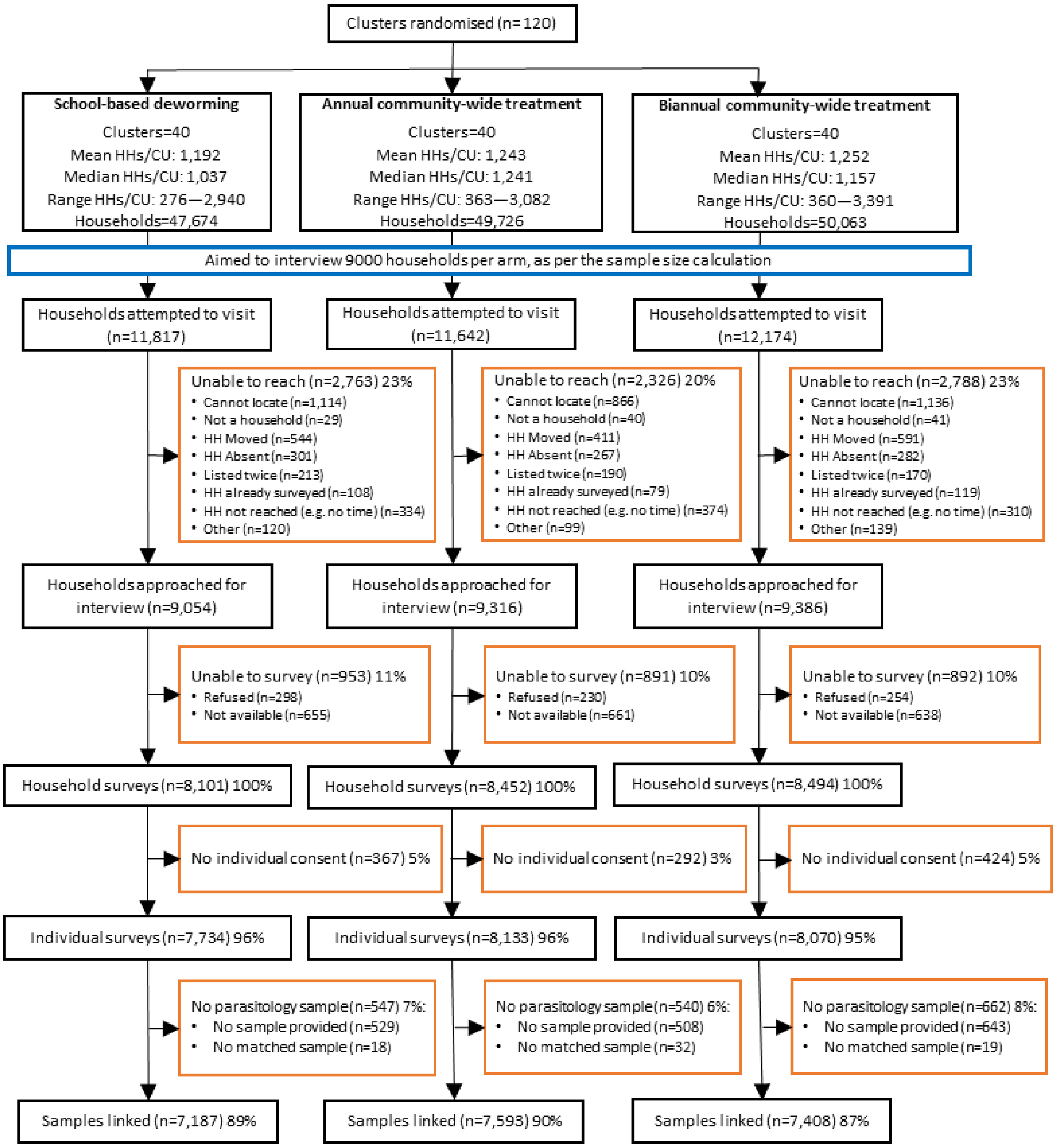

HH; household. CU; community unit

Web Table 2: Characteristics of the study population included in the 12-month assessment survey.
Data were collected during a household-based cross-sectional survey conducted from March-May 2016.

	School-based deworming		Annual community-wide treatment		Biannual community-wide treatment	
	**Households surveyed (N = 8712)**	**Participants with STH data (N = 8105)**	**Households surveyed (N = 9012)**	**Participants with STH data (N = 8449)**	**Households surveyed (N = 8874)**	**Participants with STH data (N = 8289)**
Male / Female	23679 (48·7%) / 24924 (51·3%)	3352 (41·4%) / 4753 (58·6%)	24332 (49·1%) / 25270 (50·9%)	3567 (42·2%) / 4882 (57·8%)	23979 (49·1%) / 24860 (50·9%)	3450 (41·6%) / 4839 (58·4%)
<5 Male / <5 Female	3469 (14·7%) / 3283 (13·2%)	356 (10·6%) / 338 (7·1%)	3574 (14·7%) / 3451 (13·7%)	358 (10·0%) / 359 (7·4%)	3709 (15·5%) / 3531 (14·2%)	390 (11·3%) / 406 (8·4%)
5-14 Male / 5-14 Female	7793 (32·9%) / 7651 (30·7%)	1426 (42·5%) / 1385 (29·1%)	8046 (33·1%) / 7780 (30·8%)	1460 (40·9%) / 1504 (30·8%)	7748 (32·3%) / 7695 (31·0%)	1368 (39·7%) / 1419 (29·3%)
15+ Male / 15+ Female	12417 (52·4%) / 13990 (56·1%)	1570 (46·8%) / 3030 (63·8%)	12712 (52·2%) / 14039 (55·6%)	1749 (49·0%) / 3019 (61·8%)	12522 (52·2%) / 13634 (54·8%)	1692 (49·0%) / 3014 (62·3%)
Household size	5 (1-24)	5 (1-24)	5 (1-22)	5 (1-22)	5 (1-25)	5 (1-25)
Asset index score	0·43 (-0·06-2·20)	0·43 (-0·06-2·20)	0·43 (-0·10-2·20)	0·43 (-0·10-2·20)	0·41 (-0·10-2·20)	0·41 (-0·10-2·20)
Living in poorest quintile	2190 (25·1%)	2021 (24·9%)	2409 (26·7%)	2247 (26·6%)	2519 (28·4%)	2337 (28·2%)
Electricity to household	898 (10·3%)	799 (9·9%)|	701 (7·8%)	637 (7·5%)	806 (9·1%)	716 (8·6%)
Owns a bicycle	2777 (31·9%)	2589 (31·9%)	2637 (29·3%)	2497 (29·6%)	2544 (28·7%)	2396 (28·9%)
Earth floor	6591 (75·7%)	6150 (75·9%)	7125 (79·1%)	6736 (79·7%)	6856 (77·3%)	6446 (77·8%)
Household toilet facility access:						
None	4566 (52·4%)	4253 (52·5%)	4677 (51·9%)	4406 (52·2%)	5061 (57·0%)	4740 (57·2%)
Shared access	1528 (17·5%)	1417 (17·5%)	1743 (19·4%)	1632 (19·3%)	1499 (16·9%)	1394 (16·8%)
Private access	2618 (30·1%)	2435 (30·0%)	2590 (28·8%)	2409 (28·5%)	2314 (26·1%)	2155 (26·0%)
Soap and water observed at toilet*	383 (10·1%)	357 (10·1%)	319 (8·1%)	298 (8·0%)	285 (8·2%)	257 (7·9%)
Toilet facility has washable slab**	2108 (54·3%)	1936 (53·6%)	1956 (48·4%)	1798 (47·5%)	2029 (56·9%)	1978 (56·3%)
Improved water source	4402 (50·5%)	4047 (49·9%)	5531 (61·4%)	5115 (60·5%)	4594 (51·8%)	4275 (51·6%)
Water source <30 minutes	6709 (77·0%)	6232 (76·9%)	7233 (80·3%)	6757 (80·0%)	6968 (78·5%)	6487 (78·3%)
	**Participants surveyed (N = 8433)**	**Participants with STH data (N = 8105)**	**Participants surveyed (N = 8766)**	**Participants with STH data (N = 8449)**	**Participants surveyed (N = 8613)**	**Participants with STH data (N = 8289)**
Attending primary school#	2635 (91·6%)	2574 (91·6%)	2738 (90·4%)	2682 (90·5%)	2562 (89·7%)	2500 (89·7%)
Observed wearing shoes	3929 (48·1%)	3900 (48·1%)	4153 (48·8%)	4114 (48·7%)	4091 (48·9%)	4049 (48·9%)
Dewormed in past year (outside study interventions):##	242 (3·0%)	242 (3.1%)	190 (2·3%)	188 (2·3%)	129 (1·6%)	127 (1·6%)
At health centre	235 (97·1%)	235 (97·1%)	178 (93·7%)	176 (93·6%)	123 (95·4%)	122 (96·1%)
Other location / source	7 (2·9%)	7 (2·9%)	12 (6·3%)	12 (6·4%)	5 (3·9%)	6 (3·9%)

Data are n (%) or median (range). *Data available for 11,205 households with toilet facilities on premises that agreed to direct observation and without missing information on soap availability (n=289). **Data available for 11,489 households with toilet facilities on premises that agreed to observation and without missing information on floor material (n=5). #Of those aged 5-14 years. ##Data available for 24,516 individuals among participants surveyed and 24,313 among participants with STH data.

Web Table 3: Characteristics of the study population included in the 24-month assessment survey.

Data were collected during a household-based cross-sectional survey conducted from March-May 2017.

	School-based deworming		Annual community-wide treatment		Biannual community-wide treatment	
	**Households surveyed (N = 8101)**	**Participants with STH data (N = 7187)**	**Households surveyed (N = 8452)**	**Participants with STH data (N = 7593)**	**Households surveyed (N = 8494)**	**Participants with STH data (N = 7408)**
Male / Female	23301 (48·9%) / 24313 (51·1%)	2955 (41·1%) / 4232 (58·9%)	24227 (49·1%) / 25157 (50·9%)	3191 (42·0%) / 4402 (58·0%)	23642 (49·2%) / 24397 (50·8%)	3115 (42·0%) / 4293 (58·0%)
<5 Male / <5 Female	3504 (15·0%) / 3305 (13·6%)	359 (12·2%) / 350 (8·3%)	3585 (14·8%) / 3522 (14·0%)	389 (12·2%) / 357 (8·1%)	3684 (15·6%) / 3487 (14·3%)	399 (12·8%) / 359 (8·4%)
5-14 Male / 5-14 Female	7759 (33·3%) / 7467 (30·7%)	1286 (43·5%) / 1309 (30·9%)	8042 (33·2%) / 7773 (30·9%)	1349 (42·3%) / 1333 (30·3%)	7593 (32·1%) / 7495 (30·7%)	1286 (41·3%) / 1278 (29·8%)
15+ Male / 15+ Female	12038 (51·7%) / 13541 (55·7%)	1310 (44·3%) / 2573 (60·8%)	12600 (52·0%) / 13862 (55·1%)	1453 (45·5%) / 2712 (61·6%)	12365 (52·3%) / 13415 (55·0%)	1430 (45·9%) / 2656 (61·9%)
Household size	6 (1-25)	6 (1-25)	6 (1-27)	6 (1-27)	6 (1-22)	6 (1-22)
Asset index score	0·49 (-0·01-2·21)	0·49 (-0·01-2·21)	0·49 (-0·01-2·21)	0·49 (-0·01-2·21)	0·45 (-0·10-2·21)	0·45 (-0·10-2·21)
Living in poorest quintile	2141 (26·4%)	1895 (26·4%)	2365 (28·1%)	2121 (27·9%)	2324 (27·4%)	1983 (26·8%)
Electricity to household	1198 (14·8%)	1039 (14·5%)	1035 (12·3%)	903 (11·9%)	1146 (13·5%)	979 (13·2%)
Owns a bicycle	2159 (26·7%)	1940 (27·0%)	2035 (24·2%)	1850 (24·4%)	2134 (25·1%)	1879 (25·4%)
Earth floor	6010 (74·2%)	5406 (75·2%)	6520 (77·4%)	5915 (77·9%)	6435 (75·8%)	5630 (76·0%)
Household toilet facility access:						
None	3584 (44·3%)	3182 (44·3%)	3518 (41·8%)	3154 (41·5%)	3838 (45·2%)	3295 (44·5%)
Shared access	2063 (25·5%)	1831 (25·5%)	2297 (27·3%)	2069 (27·3%)	2234 (26·3%)	1967 (26·6%)
Private access	2453 (30·3%)	2173 (30·2%)	2607 (31·0%)	2369 (31·2%)	2420 (28·5%)	2145 (29·0%)
Soap and water observed at toilet*	615 (15·2%)	516 (14·3%)	682 (15·4%)	623 (15·5%)	613 (14·8%)	537 (14·6%)
Toilet facility has washable slab**	2264 (56·0%)	1972 (54·8%)	2208 (49·7%)	1977 (49·1%)	2218 (53·6%)	1930 (52·6%)
Improved water source	4468 (55·2%)	3913 (54·5%)	5209 (61·9%)	4671 (61·5%)	4696 (55·3%)	4117 (55·6%)
Water source <30 minutes	5921 (73·1%)	5246 (73·0%)	6177 (73·3%)	5540 (73·0%)	6485 (76·4%)	5640 (76·1%)
	**Participants surveyed (N = 7734)**	**Participants with STH data (N = 7187)**	**Participants surveyed (N = 8133)**	**Participants with STH data (N = 7593)**	**Participants surveyed (N = 8070)**	**Participants with STH data (N = 7408)**
Attending primary school#	2492 (91·8%)	2388 (92·0%)	2564 (91·6%)	2457 (91·6%)	2427 (90·2%)	2312 (90·2%)
Observed wearing shoes	3249 (45·1%)	3237 (45·0%)	3503 (45·9%)	3483 (45·9%)	3375 (45·4%)	3365 (45·4%)
Dewormed in past year (outside study interventions):##	439 (6·3%)	437 (6.3%)	506 (6·9%)	505 (6·9%)	392 (5·5%)	390 (5·5%)
At health centre	402 (91·6%)	400 (91·5%)	410 (81·0%)	409 (81·0%)	310 (79·1%)	309 (79·2%)
Other location / source	37 (8·4%)	37 (8·5%)	96 (19·0%)	96 (19·0%)	82 (20·9%)	81 (20·8%)

Data are n (%) or median (range). *Data available for 12,626 households with toilet facilities on premises that agreed to direct observation. **Data available for 12,622 households with toilet facilities on premises that agreed to observation and without missing information on floor material (n=4). #Of those aged 5-14 years. ##Data available for 21,528 individuals among participants surveyed and 21,464 among participants with STH data.

Web Appendix 3: Additional results (secondary outcomes)

In the following pages we present results trial secondary outcomes: impact on hookworm and *T. trichiura* infection intensity; impact of treatment strategies on hookworm infection prevalence and intensity by pre-defined sub-group; treatment coverage of school-based deworming, as measured using coverage surveys conducted in school. All methods are reported in the accompanying manuscript.

Web Table 4 Effect of annual and biannual community-wide treatment relative to school-based deworming on intensity (as measured by eggs per gram (EPG)) of hookworm and *T. trichiura* after one and two years of intervention.

	Mean intensity in EPG (95% CI)	Absolute EPG change from baseline (95% CI)	Unadjusted incidence risk ratio* (95% CI)	p value	Adjusted** incidence risk ratio* (95% CI)	p value
**Hookworm**						
**Year 1**						
School-based deworming	129 (86,171)	-41 (-91,9)	1		1	
Annual community-wide treatment	72 (48,95)	-103 (-169,-38)	0·60 (0·42-0·85)		0·53 (0·37-0·75)	
Biannual community-wide treatment	68 (45,92)	-90 (-132,-48)	0·54 (0·36-0·79)	0.002	0·52 (0·37-0·72)	<0·001
**Year 2**						
School-based deworming	129 (86,172)	-41 (-94,13)	1		1	
Annual community-wide treatment	45 (29,61)	-130 (-191,-68)	0·39 (0·27-0·54)		0·50 (0·32-0·78)	
Biannual community-wide treatment	39 (23,55)	-119 (-162,-77)	0·30 (0·19-0·48)	<0·001	0·30 (0·19-0·46)	<0·001
T. trichiura						
**Year 1**						
School-based dewormingq	20 (6,35)	8 (-6,22)	1		1	
Annual community-wide treatment	7 (3,10)	-2 (-7,4)	0·45 (0·21-0·94)		0·76 (0·39-1·48)	
Biannual community-wide treatment	16 (2,30)	-13 (-39,13)	0·87 (0·40-1·90)	0·08	0·69 (0·35-1·33)	0·54
**Year 2**						
School-based deworming	23 (7,40)	11 (-5,26)	1		1	
Annual community-wide treatment	9 (4,13)	0 (-5,6)	0·58 (0·27-1·28)		0·53 (0·28-1·03)	
Biannual community-wide treatment	19 (5,34)	-10 (-36,16)	0·87 (0·36-2·12)	0·31	0·72 (0·38-1·40)	0·217

EPG; eggs per gram of faeces. * Zero-inflated negative binomial regression model, inflating for sex, age (2-4yrs, 5-14 yrs, >14 yrs) and baseline cluster prevalence. **Adjusted for stratification factors (sub-county, baseline cluster prevalence, cluster size), urban/rural status and baseline cluster mean socio-economic status and access to sanitation and improved water-source. Sampling was conducted at randomly selected households, selecting one household member to participate at random.

Web Table 5 Effect of annual and biannual community-wide treatment relative to annual school-based deworming on prevalence of hookworm after twenty-four months of intervention in key sub-groups

Demographic and socio-economic sub-group	Unadjusted Risk Ratio* (95% CI)	p value #	Adjusted** Risk Ratio* (95% CI)	p value #
**Annual community-wide treatment**				
Females	0·50 (0·34-0·73)		0·54 (0·43-0·69)	
Males	0·69 (0·49-0·97)		0·74 (0·59-0·94)	
**Biannual community-wide treatment**				
Females	0·42 (0·29-0·60)		0·45 (0·36-0·55)	
Males	0·49 (0·36-0·68)	0·03	0·53 (0·43-0·64)	0·03
**Annual community-wide treatment**				
Not attending school (2-14 years)	0·56 (0·33-0·94)		0·60 (0·40-0·90)	
Attending school (2-14 years)	0·66 (0·42-1·02)		0·70 (0·52-0·94)	
Adult (15+ years)	0·56 (0·41-0·77)		0·61 (0·50-0·74)	
**Biannual community-wide treatment**				
Not attending school (2-14 years)	0·55 (0·34-0·89)		0·58 (0·40-0·86)	
Attending school (2-14 years)	0·51 (0·33-0·78)		0·54 (0·41-0·70)	
Adult (15+ years)	0·42 (0·31-0·57)	0·25	0·44 (0·37-0·53)	0·26
**Annual community-wide treatment**				
Poorest quintile	0·61 (0·42-0·89)		0·66 (0·51-0·86)	
Mid three quintiles	0·59 (0·42-0·83)		0·62 (0·51-0·76)	
Least poor quintile	0·53 (0·34-0·83)		0·57 (0·39-0·84)	
**Biannual community-wide treatment**				
Poorest quintile	0·42 (0·30-0·59)		0·45 (0·35-0·56)	
Mid three quintiles	0·47 (0·34-0·66)		0·50 (0·41-0·60)	
Least poor quintile	0·50 (0·31-0·81)	0·74	0·53 (0·37-0·77)	0·68
**Annual community-wide treatment**				
No / shared toilet	0·62 (0·43-0·90)		0·68 (0·52-0·88)	
Private toilet	0·60 (0·40-0·88)		0·63 (0·50-0·79)	
**Biannual community-wide treatment**				
No / shared toilet	0·49 (0·35-0·68)		0·52 (0·42-0·64)	
Private toilet	0·42 (0·29-0·62)	0·51	0·45 (0·37-0·55)	0·43
**Annual community-wide treatment**				
Remote household +	0·59 (0·42-0·83)		0·64 (0·52-0·79)	
Accessible household ++	0·59 (0·36-0·97)		0·61 (0·39-0·96)	
**Biannual community-wide treatment**				
Remote household +	0·45 (0·32-0·63)		0·49 (0·41-0·59)	
Accessible household ++	0·48 (0·29-0·82)	0·97	0·46 (0·30-0·71)	0·95

* Relative to hookworm infection in school-based deworming arm **Adjusted for stratification factors (sub-county, baseline cluster prevalence, cluster size), urban/rural status and baseline cluster mean socio-economic status, access to sanitation and access to improved water. # p value for interaction term. + Defined as >4km from a major road; ++ defined as ≤ 4km from major road. Sampling was conducted at randomly selected households, selecting one household member to participate at random.

Web Table 6 Effect of annual and biannual community-wide treatment relative to annual school-based deworming on hookworm infection intensity after twenty-four months of intervention in key sub-groups

Demographic and socio-economic sub-group	Unadjusted Intensity Rate Ratio* (95% CI)	p value #	Adjusted** Intensity Rate Ratio* (95% CI)	p value #
**Annual community-wide treatment**				
Females	0.25 (0.16-0.40)		0.30 (0.20-0.46)	
Males	0.50 (0.32-0.80)		0.69 (0.39-1.21)	
**Biannual community-wide treatment**				
Females	0.36 (0.18-0.74)		0.35 (0.18-0.66)	
Males	0.23 (0.14-0.40)	0.02	0.24 (0.15-0.39)	0.003
**Annual community-wide treatment**				
Not attending school (2-14 years)	1.07 (0.35-3.50)		1.59 (0.48-5.24)	
Attending school (2-14 years)	0.39 (0.21-0.72)		0.51 (0.29-0.94)	
Adult (15+ years)	0.34 (0.30-0.47)		0.43 (0.29-0.65)	
**Biannual community-wide treatment**				
Not attending school (2-14 years)	0.31 (0.14-0.67)		0.48 (0.23-0.98)	
Attending school (2-14 years)	0.25 (0.13-0.48)		0.27 (0.20-0.46)	
Adult (15+ years)	0.30 (0.18-0.52)	0.27	0.28 (0.17-0.47)	0.15
**Annual community-wide treatment**				
Poorest quintile	0.41 (0.25-0.67)		0.48 (0.28-0.83)	
Mid three quintiles	0.39 (0.26-0.60)		0.58 (0.35-0.96)	
Least poor quintile	0.21 (0.07-0.62)		0.32 (0.12-0.84)	
**Biannual community-wide treatment**				
Poorest quintile	0.15 (0.09-0.28)		0.18 (0.10-0.33)	
Mid three quintiles	0.33 (0.19-0.57)		0.31 (0.19-0.51)	
Least poor quintile	1.07 (0.22-5.22)	0.005	1.05 (0.26-4.17)	0.02
**Annual community-wide treatment**				
No / shared toilet	0.51 (0.32-0.83)		0.62 (0.35-1.11)	
Private toilet	0.30 (0.19-0.48)		0.39 (0.23-0.64)	
**Biannual community-wide treatment**				
No / shared toilet	0.32 (0.16-0.62)		0.32 (0.18-0.57)	
Private toilet	0.28 (0.15-0.53)	0.25	0.23 (0.13-0.40)	0.36
**Annual community-wide treatment**				
Remote household +	0.39 (0.27-0.57)		0.50 (0.32-0.80)	
Accessible household ++	0.38 (0.20-0.71)		0.54 (0.22-1.29)	
**Biannual community-wide treatment**				
Remote household +	0.33 (0.20-0.55)		0.33 (0.21-0.53)	
Accessible household ++	0.13 (0.07-0.24)	0.04	0.16 (0.08-0.32)	0.21

* Relative to hookworm infection in school-based deworming arm ** Zero-inflated negative binomial regression model, inflating for sex, age (2-4yrs, 5-14 yrs, >14 yrs) and baseline cluster prevalence. # Adjusted for stratification factors (sub-county, baseline cluster prevalence, cluster size), urban/rural status and baseline cluster mean socio-economic status and access to sanitation and improved water-source. ## p value for interaction term. .+ Defined as >4km from a major road; ++ defined as ≤ 4km from major road. Sampling was conducted at randomly selected households, selecting one household member to participate at random.

Web Table 7: Treatment coverage during school-based deworming, as measured during treatment coverage surveys conducted in schools.

School catchments do not align to study cluster (community unit) boundaries, as one school may serve several community units, and conversely one community unit may contain several schools. Treatment data are therefore stratified by study arm based on reported village of residence for each student (with multiple villages in a cluster). If this information was unavailable, students were assigned to the nearest community unit to the school.

	**Round 1 - 2015**	**Round 3 - 2016**
Schools surveyed	454	469
School participated in NSBDP	453 (99·8%)	469 (100·0%)
Students sampled	21624	21435
Student/respondent available	20833 (96·3%)	20662 (96·4%)
Student/respondent assents	20784 (96·1%)	20554 (95·9%)
Student eligible*	14731 (68·1%)	14189 (66·2%)
Population treatment coverage#	87·8% (86·5-89·1%)	87·9% (85·7-90·0%)
Treatment coverage by study-arm#:		
School-based deworming (SBD)	88·3% (86·1-90·6%)	86·2% (82·0-90·4%)
Annual community-wide Treatment (CWT)	88·2% (86·2-90·1%)	89·1% (85·2-93·0%)
Biannual community-wide Treatment (CWT)	86·8% (84·0-90·0%)	88·4% (85·5-91·3%)

*Eligible if aged 5-14 years and reported to live in study communities

Standard errors adjusted for clustering by community unit (n=120)

Web Appendix 3: Safety Report across all four rounds of implementation.

There were three reported severe adverse events (SAEs), which are summarised below. Two of these were in the biannual community-wide treatment arm, and one in a cluster that was not included in the TUMIKIA trial (one of the ten excluded community units). None of the SAEs were considered to be related to albendazole, the treatment given out in the TUMIKIA intervention arms.

Web Table 8: Severe adverse events (SAEs) reported to trial data & safety monitoring board between April 2015 and May 2017.

School-based deworming arm	Annual community-wide treatment arm	Biannual community-wide treatment arm
No SAEs	No SAEs	Female, 6 years old, part of the school deworming exercise in Msambweni-Lungalunga.<br><br>Participant died 29/05/2016.<br><br>Stated cause of death – severe malaria
		Female, 62 years old.<br><br>Seen in hospital on 31/10/2016.<br><br>X-ray for bone formation / development. Side effects not related to ALB or DEC administration.

Note: One SAE was reported in Kilolapwa, however this cluster was not included in the TUMIKIA trial. Two year old male, admitted to hospital on 29/10/2016 for side effects due to diethylcarbamazine (DEC) overdose and discharged the next morning.

Web Appendix 4: Details of Intervention Costing

Cost data were collected for the third and fourth rounds of community-based mass deworming treatment, representing one full year of implementation. The costing was conducted from a provider's perspective and is presented in two ways: study-implemented (i.e. inclusive of all costs incurred that were directly related to implementation) and a routine-implemented scenario (i.e. all costs likely to be incurred if implemented as a stand-alone programme). Costs were annualized at a 3% discount rate, and are presented in constant 2016 United States Dollars (USD).

Both financial and economic costs were collected. Financial costs represent any expenditure incurred during the implementation of the intervention. Economic costs indicate the full value of all resources used to implement the intervention, regardless of whether they incurred a financial cost. All research costs related to the TUMIKIA trial are excluded (e.g. resources necessary to conduct assessment surveys). Where a cost was incurred across both research and implementation (e.g. staff employed on an annual contract to implement all components of the trial), only the proportion of that resource that can be directly attributed to implementation is used to define the cost. Where possible this is derived from study records (e.g. procurement records, time-sheets) or estimated through interviews with members of the study team

All costs were attributed to the unit of implementation (i.e. the community unit). Where costs were incurred at a higher administrative level (e.g. sub-county supervision) or across multiple clusters (e.g. two community units trained together) these were attributed equally between the clusters directly related to the cost. Where possible, costs were directly extracted from financial records of each activity and reflect actual expenditure at each treatment round. Costs were categorised by both activity and resource type.

Personnel: For non-study staff (e.g. government employees), the financial costs of involvement in implementation consisted of per diems, whereas the economic costs included both these per diems and a share of the monthly costs to their employer of employing them (i.e. salary, fringe benefits, taxes, etc.) that reflected the share of their overall working time spent on implementing the intervention. The salary of each national, county and sub-county level officer was estimated by interviewing a sample of individuals and using the mid-point of the modal salary band at each administrative level. Each CHA was interviewed and the mid-point of the reported salary band used to estimate their salary. As the majority of CHVs reported subsistence or smallholder farming as their primary occupation, the national minimum wage for agricultural work by an unskilled worker was used to value their time, and taken as the cost of employment where it exceeded the value of any per-diem provided. The cost of employing all research staff (KEMRI and LSHTM) includes their salary, fringe benefits and statutory taxes, was taken directly from employer's financial records.

Commodities: The study drug (albendazole) was donated free of charge by GSK through WHO to the Government of Kenya. The financial cost attributed to the study drug (e.g. central handling and storage) was calculated as 3% of the list price based on information provided by KEMSA (Kenya Medical Supplies Authority), whilst the additional economic cost is taken as the remaining 97% of the list price. Drug costs reflect the total quantity required to implement the intervention and as such includes both drugs "used" (e.g. administered or wasted) and "not used" (e.g. safety/buffer stocks).

Assets: The equivalent annual cost of Ministry of health, county or sub-county owned vehicles and other equipment (e.g. laptops, printers & smartphones) used during implementation was calculated using site-specific estimates of the total import cost, lifespan, insurance and routine maintenance costs and a 3% discount rate applied. An equivalent daily (unit) cost of vehicles was calculated using an estimate of 250 days use per year. Daily vehicle usage was extracted from operational records (e.g. log books) and reported usage.

Other: The costs of vehicle hire, venue hire (e.g. for training or office space) and local transport (e.g. taxis) were extracted from financial records. All hired vehicles were used for intervention delivery activities that were specific to the trial (e.g. to transport research staff to supervise trainings), and so are included in the main cost estimates, but excluded for the routine implementation scenario.

Sensitivity analysis

Univariate sensitivity analysis was conducted on selected key parameters, including the shadow price of albendazole, per diems given to CHVs, the discount rate, and the exchange rate. The market price of an albendazole tablet (400mg) was derived from the KEMSA list price in January 2017 ($0.025) and most recent WHO estimates for programme planning ($0.02-$0.03). The lower bound of CHV per diem was informed by Kenyan national policy guidelines, which recommend a monthly stipend of KES 2000 is paid to CHVs (approximately equivalent to KES 500 for an 8 day community-wide

treatment campaign). The upper bound of CHV per diem for implementing community-wide treatment was based on the highest daily rate paid to CHVs during the study (KES 800 for attending a 1-day training).

Doubling of the market price of albendazole from $0.025 to $0.05 per tablet increased economic costs to $0.93 per person treated for delivery alongside school-based distribution (+7%) and to $0.81 per person treated for delivery to all ages (+6%). The per diem given to CHVs was varied between $0.62 per day (KES 500 for all 8 days) and $7.93 per day (KES 800 per day), resulting in $0.75-0.95 per person treated for delivery alongside school-based distribution (-15% to +9%) and $0.66-0.83 per person treated for delivery to all ages (-14% to +9%). Currency exchange and discount rate had negligible impact.

Routine Implementation Scenario

The routine implementation scenario removes any costs that would no longer be incurred if community-wide treatment was implemented as an independent programme (i.e. outside the context of the TUMIKIA) while still to a similar level of fidelity as during the study. No changes are made to the core aspects of the intervention, such as planning, drug distribution, drug administration and supervision cost centres; the removed costs relate primarily to the per diem or salary and transport costs of study personnel employed to support the implementation of CBD:

- **Training:** Study staff supervision of CHA and CHV trainings are removed, with training wholly the responsibility of the facilitators (CHAs and/or sub-county level staff) and routine supervisors (sub-county and/or county levels staff).
- **Sensitisation:** Study staff supervision of pre-implementation meetings held with control arm clusters are removed, with sensitisation wholly the responsibility of the facilitators (CHAs and Village headman) and routine supervisors (sub-county level staff).
- **Implementation monitoring:** The number of CHV spotchecks during CBD and post-monitoring coverage surveys conducted by study staff are reduced by 75%. Study staff do not conduct data entry of treatment registers to generate updated household listings for the following treatment round.
- **Routine reporting:** Costs of transporting treatment registers by the study team from clusters to sub-county or county offices following completion of MDA are removed. Treatment booklets are expected to be transported as-hoc using sub-county of county vehicles when available.
- **Central administration:** All international research staff are removed, with an increase in the number of locally-employed staff wholly responsible for implementation.

Web Table 9: Cost matrix describing breakdown of costs included within each of the cost categories, highlighting implementation costs specific to the trial context that are removed for the implementation scenario.

Cost category:	Description:
Planning	• One-day review and planning meeting with county and sub-county level staff.<br><br>**Personnel:** Per-diems and unpaid time (County & sub-county officers)<br>**Commodities:** Meals & refreshments, fuel<br>**Utilities:** Venue hire, vehicle hire<br><br>**Implementation costs specific to trial context:** Vehicle hire & fuel for senior study officers to facilitate a planning meeting for MDA. These costs are removed in the routine implementation scenario, and as such the planning meeting would be expected to be facilitated by member(s) of county-level staff.
Training	• One-day CHA training meetings in each sub-county, each facilitated by two sub-county officers<br>• One day training of CHVs in each community health unit, each facilitated by CHA(s)<br><br>**Personnel:** Per-diems and unpaid time (County officers, sub-county officers & CHAs)<br>**Commodities:** Stationary, Meals & refreshments, Fuel<br>**Assets:** CHMT/SCHMT vehicle usage<br>**Utilities:** Venue hire, vehicle hire<br><br>**Implementation costs specific to trial context:** Vehicle hire and fuel for study officers to facilitate CHA trainings and supervise CHV training sessions are removed from the routine implementation scenario. CHA trainings would continue to be facilitated by sub-county level staff, and CHAs would be wholly responsible for facilitating CHV training.
Community sensitisation	• Half-day planning meeting in each sub-county facilitated by senior study officers with ward administrators and chiefs<br>• Half day sensitisation meeting in each sublocation (equivalent to between 1-3 study clusters) facilitated by study officers and CHA(s) with respective village administrators and village elders.<br><br>**Personnel:** Per-diems (County officers, sub-county officers, Ward administrators, Chiefs, Assistant chiefs, Village administrators, Village elders & CHAs) and unpaid time (Village elders and CHAs)<br>**Commodities:** Airtime, Fuel allowances<br>**Assets:** CHMT/SCHMT vehicle usage<br>**Utilities:** Vehicle hire<br><br>**Implementation costs specific to trial context:** Vehicle hire and fuel for study officers to facilitate sensitisation planning meeting and sublocation-level meetings. These costs are removed in the routine implementation scenario, with facilitation of sensitisation meetings continuing to be conducted by Village administrators and CHAs.
Drug transport	• Transport of drugs from national to county stores, and then from county to health facility<br><br>**Personnel:** Per-diems (County officers, sub-county officers, drivers) and unpaid time (CHAs)<br>**Commodities:** Airtime, Fuel allowances & fuel<br>**Assets:** CHMT/SCHMT vehicle usage<br>**Utilities:** Vehicle hire<br><br>**Implementation costs specific to trial context:** Vehicle hire and fuel for two additional vehicles to support drug transport from county to health facilities. These costs are removed in the routine implementation scenario, and would be expected to be combined with routine drug distribution from county-level stores.
Drug administration	• Drug administration conducted door-to-door by CHVs over eight days.<br><br>**Personnel:** Per-diems (CHVs)<br>**Commodities:** Albendazole, Treatment handbook & job-aids, t-shirts, bags<br><br>This cost centre has no implementation costs specific to the trial context.
Supervision	• CHAs supervise CHVs by field observation and over the phone during drug administration<br>• County and sub-county officers provide oversight and ad-hoc supervision of CHAs and CHVs during drug administration<br>• National-level officer conducts three-day site visit during drug administration<br><br>**Personnel:** Per-diems (National, county & sub-county officers)<br>**Commodities:** Domestic flight tickets, lunch and fuel allowances, airtime, t-shirts<br>**Assets:** CHMT/SCHMT vehicle usage<br><br>This cost centre has no implementation costs specific to the trial context.
Implementation monitoring	• 'Spotchecks' of CHVs administering treatment at household level conducted by study officers<br>• Coverage survey in random sample of households after treatment campaign is completed conducted by study officers<br>• Data entry of treatment registers to update household listings in preparation for next treatment round conducted by study officers

	**This entire cost centre is specific to the trial context:** Per-diems (research staff), vehicle hire and fuel to transport study officers during spotchecks and coverage survey, and smartphones for data entry. In the routine implementation scenario, spotchecks and coverage surveys are scaled back by 75%, but are expected to be implemented in a similar fashion to the study and as such all ingredients (e.g. daily vehicle hire, data collector per-diem rates) remain the same individual costs as during the trial. Household listings are not transcribed between rounds of MDA to generate updated lists, and are instead expected to be maintained by CHVs as part of their routine activities.
Routine reporting	• One-day consolidation of CHV treatment summaries by CHAs<br>• Four-day entry of treatment records in routine health reporting system by HIROs at sub-county level<br><br>**Personnel:** Per-diems (County officers, sub-county officers & HIROs)<br>**Commodities:** Fuel<br>**Assets:** Laptops for data entry<br>**Utilities:** Vehicle hire<br><br>**Implementation costs specific to trial context:** Vehicle hire and fuel for one vehicle per sub-county for three days to collect treatment booklets. These costs are removed in the routine implementation scenario, and would be expected to be combined ad-hoc with county or sub-county vehicles moving between health facilities and sub-county offices.
Central administration	• Salaries and fringe benefits of both international (LSHTM) and local (KEMRI) staff for the implementation period including trial coordinator, project manager, research fellow, research assistant and six project associates.<br>• Costs related to senior GoK personnel are included within planning and supervision cost centres<br>• Travel and subsistence costs of international staff<br>• Office rental, equipment, communications, stationary and local transport.<br><br>**Personnel:** Salaries and fringe benefits (LSHTM and KEMRI staff)<br>**Commodities**: Travel and subsistence of international staff, airtime, stationary and refreshments<br>**Assets:** Laptop computers and printers<br>**Utilities:** Office rental and local transport<br><br>**Implementation costs specific to trial context:** Salaries, fringe benefits, travel and subsistence of international staff. These costs are removed in the routine implementation scenario and replaced with the equivalent number of local INGO staff, based on KEMRI rates. The implementation period is also reduced from six to four weeks (reflecting the removal of all study-related activities) and therefore the quantity of all ingredients in this cost category is reduced proportionally.

Web Figure 5: Cost of albendazole distribution immediately following a school-based strategy (round 3) by resource type and activity

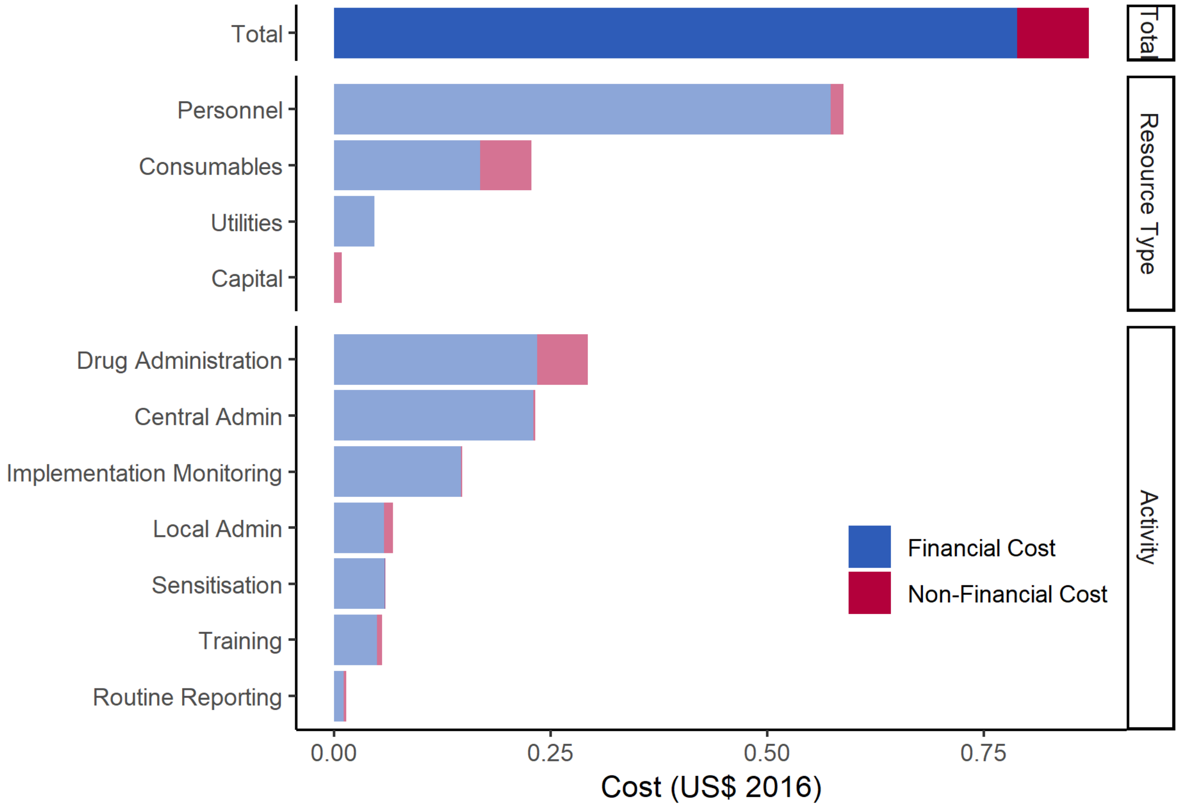

Web Figure 6: Heterogeneity of cost per person treated by community-wide treatment by trial arm and round, with variation by cluster. The box range represents the 25-75th percentile range. The line range extends to the largest value no further than 1.5 times this inter-quartile range. Outliers are represented as points.

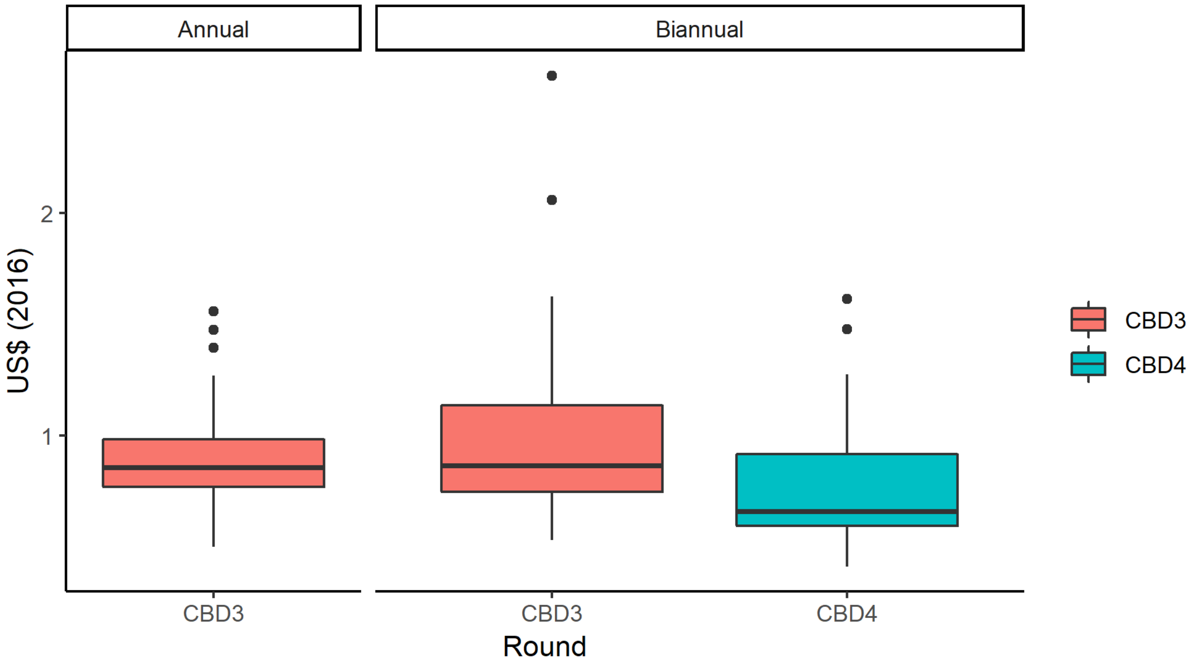

Web Table 10: Financial cost of community-based drug administration by trial arm and round. Round 3 was the third round of treatment, and was conducted immediately after school-based deworming targeting all community members over two years who were not treated in school. Round 4 was the fourth round of treatment conducted six months later, and targeted all community members.

Costs presented in constant 2016 USD.

		Whole Trial Estimates			Variation Across Clusters		
**Trial arm**	**Round**	**Number of treatments administered**	**Cost per treatment administered**	**Total cost**	**Number of treatments administered (range)**	**Median cluster cost per treatment administered (range)**	**Median cost per cluster (range)**
Annual community-wide treatment	3	120,083	$ 0·75	$ 90,553	2,914 (1,303-6,075)	$ 0·77 (0·48-1·46)	$ 2,255 (1,898-2,898)
	3 and 4	283,409	$ 0·69	$ 194,436	7,246 (2,121-14,302)	$ 0·67 (0·43-1·71)	$ 4,829 (3,622-6,154)
Biannual community-wide treatment	3	115,279	$ 0·79	$ 90,550	2,822 (689-5,892)	$ 0·80 (0·50-2·41)	$ 2,269 (1,661-2,935)
	4	168,130	$ 0·62	$ 103,886	4,206 (1,339-8,410)	$ 0·61 (0·38-1·46)	$ 2,560 (1,961-3,219)

Web Table 11: Economic cost of community-based drug administration by trial arm and round. Round 3 was the third round of treatment, and was conducted immediately after school-based deworming targeting all community members over two years who were not treated in school. Round 4 was the fourth round of treatment conducted six months later, and targeted all community members.

Costs presented in constant 2016 USD.

		**Whole Trial Estimates**			**Variation Across Clusters**		
**Trial arm**	**Round**	**Number of treatments administered**	**Cost per treatment administered**	**Total cost**	**Number of treatments administered (range)**	**Median cluster cost per treatment administered (range)**	**Median cost per cluster (range)**
Annual community-wide treatment	3	120,083	$ 0·84	$ 100,413	2,914 (1,303-6,075)	$ 0·86 (0·55-1·56)	$ 2,502 (2,031-3,358)
	3 and 4	283,409	$ 0·76*	$ 214,589	7,246 (2,121-14,302)	$ 0·74 (0·49-1·85)	$ 5,385 (3,919-6,961)
Biannual community-wide treatment	3	115,279	$ 0·87	$ 100,454	2,822 (689-5,892)	$ 0·90 (0·57-2·62)	$ 2,535 (1,815-3,331)
	4	168,130	$ 0·68	$ 114,135	4,206 (1,339-8,410)	$ 0·68 (0·43-1·58)	$ 2,846 (2,117-3,630)

Web Table 12: Economic and financial cost of routine intervention scenario for community-wide treatment. The scenario for trial areas removes any costs that would no longer be incurred if community-wide treatment was implemented as an independent programme but within the same study clusters. The whole county scenario expands this programme to treat all community units in Kwale county. Round 3 was the third round of treatment, and was conducted immediately after school-based deworming targeting all community members over two years who were not treated in school. Round 4 was the fourth round of treatment conducted six months later, and targeted all community members over two years.

Costs presented in constant 2016 USD.

		Financial cost	Economic cost	Treated	Number of community units included	Financial cost per treatment	Economic cost per treatment	Difference in economic cost per treatment (relative to trial)	% Change (relative to trial)
**Round 3**	**Trial implementation**	$182,325	$202,222	235,362	80	$0.77	$0.86		
	**Routine scenario, trial areas**	$101,926	$120,550	235,362	80	$0.43	$0.51	$0.35	40%
	**Routine scenario, whole county**	$142,011	$167,927	353,043	130	$0.40	$0.48	$0.04	4%
**Round 4**	**Trial implementation**	$104,587	$114,905	168,130	40	$0.62	$0.68		
	**Routine scenario, trial areas**	$71,867	$84,588	168,130	40	$0.43	$0.50	$0.18	26%
	**Routine scenario, whole county**	$143,941	$168,778	504,390	130	$0.29	$0.33	$0.17	25%

Web Appendix 6: Data Sharing Statement

Will individual participant data be available (including data dictionaries)?

Yes

What data in particular will be shared?

Individual participant data that underlie the impact assessment results reported in this article, after de-identification (text, tables, figures, and appendices)

What other documents will be available?

Study protocol, statistical analysis plan, informed consent form, analytic code

When will data be available (start and end dates)?

Immediately following publication; no end date.

With whom?

Researchers who provide a methodologically sound proposal.

For what types of analyses?

To achieve aims in the approved proposal.

By what mechanism will data be made available?

Proposals should be directed to rachel.pullan@lshtm.ac.uk; to gain access, data requestors will need to sign a data access agreement.

Web Appendix References

1. Njenga SM, Kanyi HM, Mutungi FM, et al. Assessment of lymphatic filariasis prior to re-starting mass drug administration campaigns in coastal Kenya. *Parasit Vectors* 2017; **10**(1): 99.
2. Ramaiah KD, Vanamail P, Pani SP, Yuvaraj J, Das PK. The effect of six rounds of single dose mass treatment with diethylcarbamazine or ivermectin on Wuchereria bancrofti infection and its implications for lymphatic filariasis elimination. *Trop Med Int Health* 2002; **7**(9): 767-74.
3. Ramzy RM, el-Setouhy M, Helmy H, et al. The impact of single-dose diethylcarbamazine treatment of bancroftian filariasis in a low-endemicity setting in Egypt. *Am J Trop Med Hyg* 2002; **67**(2): 196-200.
4. Olsen A. Efficacy and safety of drug combinations in the treatment of schistosomiasis, soil-transmitted helminthiasis, lymphatic filariasis and onchocerciasis. *Trans R Soc Trop Med Hyg* 2007; **101**: 747–58.
